# Clinical Work-Integrating Care in Current Practice: A Scoping Review

**DOI:** 10.1007/s10926-023-10143-1

**Published:** 2023-11-15

**Authors:** Lana Kluit, Coen A. M. van Bennekom, Annechien Beumer, Maayke A. Sluman, Angela G. E. M. de Boer, Astrid de Wind

**Affiliations:** 1 Department of Public and Occupational Health, Amsterdam UMC Location AMC, University of Amsterdam, Meibergdreef 9, 1105 AZ Amsterdam, The Netherlands; 2 Amsterdam Public Health Research Institute, Societal Participation and Health, Amsterdam, The Netherlands; 3 Research and Development, Heliomare Rehabilitation Centre, Wijk aan Zee, The Netherlands; 4 Upper Limb Unit Department of Orthopedic Surgery, Amphia Hospital, Breda, The Netherlands; 5 Department of Cardiology, Jeroen Bosch Hospital, Den Bosch, The Netherlands; 6 Cancer Center Amsterdam, Cancer Treatment and Quality of Life, Amsterdam, The Netherlands

**Keywords:** Return to work, Clinical decision-making, Occupational health, Physician–patient relations, Systematic review

## Abstract

**Purpose:**

Clinical work-integrating care (CWIC) refers to paying attention to work participation in a clinical setting. Working patients may benefit from CWIC. The purpose of this study is to explore the extent and nature to which medical specialists provide CWIC and what policies and guidelines oblige or recommend specialists to do.

**Methods:**

A scoping review was conducted. The databases MEDLINE, EMBASE, Psychinfo, CINAHL, and Web of Science were searched for studies on the extent and nature of CWIC and supplemented by gray literature on policies and guidelines. Six main categories were defined a priori. Applying a meta-aggregative approach, subcategories were subsequently defined using qualitative data. Next, quantitative findings were integrated into these subcategories. A separate narrative of policies and guidelines using the same main categories was constructed.

**Results:**

In total, 70 studies and 55 gray literature documents were included. The main findings per category were as follows: (1) collecting data on the occupation of patients varied widely; (2) most specialists did not routinely discuss work, but recent studies showed an increasing tendency to do so, which corresponds to recent policies and guidelines; (3) work-related advice ranged from general advice to patient–physician collaboration about work-related decisions; (4) CWIC was driven by legislation in many countries; (5) specialists sometimes collaborated in multidisciplinary teams to provide CWIC; and (6) medical guidelines regarding CWIC were generally not available.

**Conclusion:**

Medical specialists provide a wide variety of CWIC ranging from assessing a patient’s occupation to extensive collaboration with patients and other professionals to support work participation. Lack of medical guidelines could explain the variety of these practices.

**Supplementary Information:**

The online version contains supplementary material available at 10.1007/s10926-023-10143-1.

## Introduction

It is important to include all patients in the labor market and stimulate their work participation [1], because working improves someone’s overall quality of life and well-being [2–4]. Furthermore, patients generally value work as it provides income, social contacts, and the ability to contribute to society [5, 6]. However, acquiring a disease can have an impact on work participation. To illustrate, 35% of the economically inactive people in the working age population have a chronic physical or mental health problem, illness, or disability, compared to 18% of economically active people [7]. Moreover, 81% of the inactive group with health problems feels limited by their health in their daily activities. Because demographic changes in Europe are leading to an aging workforce, more people of working age are prone to acquire a disease or injury [8, 9].

As a measure to stimulate work participation, it could be beneficial for all hospital-based healthcare professionals to be more aware of this domain so they can support their patients within the boundaries of their professions [10]. Medical specialists are in the unique position to leverage their expert knowledge of determinants of health to positively contribute to a patient’s socioeconomic needs—for example, with regard to work participation [11, 12]. Many physicians nowadays are being educated to be more aware of this position they hold as health advocate [13, 14]. Physician awareness of work participation needs can aid in early detection of problems with work participation to prevent or reduce work loss due to disease [15]. Moreover, if a medical specialist is aware of a patient’s occupation, the specialist can also adapt the treatment plan accordingly and understand non-adherence to a treatment plan when work participation interferes with the medical treatment [16]. These are just some examples of support for work participation within the context of hospital-based care.

Providing support for work participation in hospital care settings is referred to as clinical work-integrating care (CWIC) [17]. Acting within the philosophy of CWIC, healthcare professionals acknowledge that work and health mutually influence each other. This implies that they pay attention both to work as a cause of disease (e.g., occupational diseases) and to the positive effects of work participation on mental and general health, as well as attention to the impact of disease or treatment on the ability to work [17]. This concept differs from the concept of work-focused healthcare, which aims to improve support for work participation within clinical healthcare [18]. Work-focused healthcare puts a strong emphasis on the responsibilities that clinical healthcare professionals have for addressing obstacles to work participation within a clinical encounter. In CWIC, on the other hand, healthcare professionals are guided by the understanding that work and health (including medical decisions) affect each other to support patients. As such, CWIC necessitates a broader perspective than merely focusing on the obstacles that patients encounter for work participation. At the same time, CWIC is less compelling by leaving out what the responsibility of the clinical healthcare professional should be in comparison with work-focused healthcare.

Within hospital-based healthcare, however, CWIC is not always applied by the medical specialist [19–22]. One reason for this may be that, in general, medical specialists take a disease-oriented focus instead of understanding work participation as a treatment goal [16, 23]. Furthermore, medical specialists can experience difficulties with the patient’s trust when their professional opinion of work ability assessment differs from the patient’s own opinion [10, 24, 25]. However, it is unknown to what extent medical specialists do provide CWIC and what the nature of this care is. It is also unclear what policy documents and medical guidelines say with respect to what medical specialists are obliged to do for the provision of CWIC. To identify the key characteristics of providing CWIC, a scoping review is most suitable [26]. Therefore, we conducted a scoping review to systematically map the extent and nature to which medical specialists provide CWIC. Furthermore, we augmented these findings with what is described in international policy documents and medical guidelines regarding the provision of CWIC. The following research questions were formulated: (1) To what extent do medical specialists provide CWIC and what is the nature of this care? (2) To what extent do policy documents and medical guidelines provide information on what medical specialists are obliged or recommended to do regarding the provision of CWIC?

## Methods

This scoping review was conducted in accordance with the methodological framework outlined by the Joanna Briggs Institute [27, 28]. The framework was used to develop our protocol, which is available upon request from the corresponding author. One of the purposes of a scoping review is to identify the key characteristics of a concept, which in our case is the provision of CWIC [26]. The PRISMA extension for scoping reviews (PRIMA-ScR) was used as a guideline for reporting [29].

### Eligibility Criteria

All studies or documents that aimed at medical specialties treating patients within the working age population (18–67 years) with primarily somatic problems in secondary or tertiary clinical health care were included. Excluded medical specialties were pediatrics, neonatology, geriatrics, psychiatry, pathology, primary health care, and occupational health care, since these specialties either do not treat patients within the working age population, focus on mental health problems, or are not situated in secondary or tertiary clinical health care. To be included, studies from scientific literature or documents from gray literature had to address the extent or nature of the provision of CWIC by medical specialists or provide information on what specialists are obliged or recommended to do regarding CWIC. For the purpose of this study, we operationalized the provision of CWIC as collecting information on occupational status; exploring work-relatedness of a disease (etiology); opening a dialogue on work (by who and when in the patient’s journey); offering support, information, or advice about work participation (including resources used by medical specialists); considering work in a treatment plan; offering work-related support regarding local regulations; and cooperating with other (medical) disciplines regarding work (interdisciplinary cooperation). To answer our first research question on the extent and nature of the provision of CWIC by medical specialists, information could either be reported by medical specialists themselves or be derived from the perspectives of patients, other healthcare professionals, or institutes in which medical specialists worked. We focused on original research and included qualitative, quantitative, and mixed-method studies. For our second research question (i.e., to what extent do policy documents and medical guidelines provide information on what medical specialists are obliged or recommended to do regarding the provision of CWIC), gray literature was explored, and information was derived from official reports on policies and vision documents to review the societal perspective and medical guidelines to review professional standards. Studies and documents had to be published in English or Dutch to be included.

### Information Sources and Search Strategy

The following bibliographic databases were searched: MEDLINE, EMBASE, Psychinfo, CINAHL, and Web of Science. We screened articles published between January 2005 and October 2021. The “PCC” mnemonic (population, concept, and context) [28], which is commonly used in the context of scoping reviews, was used to structure the search strategy. The search strategy can be found in Supplementary file 1. The search results were imported in EndNote to de-duplicate the dataset [30, 31]. After de-duplication, the results were exported into the online systematic review tool Rayyan Systems Inc. (rayyan.ai) for the selection process.

The scientific database search was supplemented with a search of gray literature from international governments and health organization websites. A list of websites is provided in Supplementary file 2.

### Selection Process

The selection of the scientific articles was performed by all authors in two steps. After screening the titles and abstracts for relevant articles, full-text screening to check articles according to the eligibility criteria was completed. All articles were screened by the first author (LK) and independently by one of the other authors. During the full-text screening, the authors encountered 57 disagreements within the 322 selected publications (17.7%). Disagreements were first discussed one-on-one between LK and the other author who had reviewed the publication. In five cases where the conflict could not be resolved one-on-one, disagreements were discussed with the whole team until consensus was reached. It was decided that articles that used the same data would be combined and reviewed as one study. The gray literature search was performed by the first author (LK). The eligibility of the documents that were identified were discussed with the team.

### Methodological Quality Appraisal

Methodological quality appraisal would not contribute to our study aims to retrieve information on the extent and nature of CWIC and what specialists are obliged to do regarding CWIC, because we expected heterogeneous results. Therefore, consistent with guidance on scoping review conduct [27, 28], we did not appraise methodological quality or risk of bias of the included studies.

### Data Extraction

From all included studies, we extracted data on study characteristics (i.e., author, year of publication, study aim, study design, study sample, and period and country of data collection) and key findings. Since the aim was to retrieve information on the extent and nature of providing CWIC within current practice, key findings—for example, use of existing programs to detect occupational disease or existing guideline—could in theory also be derived from introduction, methods, and discussion sections of the studies. From all included policies and guidelines, we extracted data on document characteristics (i.e., title, source, and year of publication) and key findings.

Data extraction was performed by LK and discussed with AdW. Two qualitative articles and ten quantitative articles were checked by AdW for inconsistencies in the data extraction. All findings from the gray literature were checked by AdW and discussed between LK and AdW until consensus on eligibility was reached. Any uncertainties related to data extraction were discussed with the whole team.

### Data Synthesis

Data synthesis was executed in four steps. Firstly, we defined six main categories, grouping the variables according to our operationalization of the provision of CWIC into logical clusters. These categories were defined to facilitate the integration of all data sources into a coherent understanding to the current and expected provision of CWIC by medical specialists. The categories were (1) exploration of work and disease work-relatedness by medical specialists, (2) discussing work-related concerns with patients, (3) the nature of the work-related advice given to patients, (4) other work-related support for patients, (5) interdisciplinary cooperation between medical specialists and other healthcare providers, and (6) medical guidelines integrating work and the use of these guidelines by medical specialists.

In the second step, we started with the synthesis of the qualitative articles, since qualitative research synthesis can create a renewed interpretation or conceptualization of a phenomenon—in our case, CWIC—that is not merely a summary of the original data [32]. For the qualitative data synthesis, we chose a meta-aggregative approach [33]. This approach consists of two procedures [33]. Firstly, all qualitative findings were labeled and organized into subcategories. Next, these subcategories were classified into the six categories defined a priori in our first step. The qualitative description of these subcategories created the core of the results.

In the third step, a data extraction form was used to separate the findings of the quantitative studies into the six main categories. These findings were then integrated with the qualitative description to support each subcategory.

Lastly, a separate description was made of the policies and guidelines to describe the extent to which medical specialists are obliged or recommended to provide CWIC. To guide this description, the six categories that had been defined a priori were again utilized.

## Results

### Study Selection

**Fig. 1:**
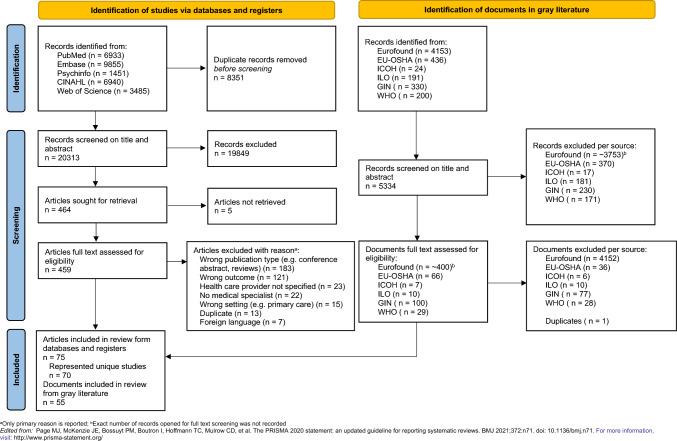
Selection process visualized with the PRISMA-ScR flow diagram

The selection process is visualized in Fig. 1. In total, we included 75 articles in this review. Several articles reported on the same dataset. After combining these studies, we included 70 unique studies. The gray literature search resulted in 55 additional policy documents and guidelines.

## The extent and nature to which medical specialists provide CWIC

### Study Characteristics

Table 1 gives an overview of the study characteristics of all 70 studies. A detailed description of each study is provided in Supplementary file 3. All studies were published in English. Almost, a third of the included studies were qualitative research (*n* = 22, of which two were mixed-methods studies) investigating the perspectives of medical specialists, patients, and other healthcare professionals (e.g., physiotherapists, nurses) or a combination of perspectives [34–56]. Most quantitative studies were cross-sectional (*n* = 39) addressing a sample of medical specialists and in some cases patients and other healthcare professionals as well [47, 57–97]. Other study designs were cohort studies (*n* = 8) [98–106], and there was one prospective longitudinal study [107]. Studies originating from the UK (*n* = 18) [35, 36, 38, 45, 49, 53, 55, 57, 58, 60, 61, 64, 78, 79, 82, 84, 86, 90] and Sweden (*n* = 13) [41, 42, 52, 59, 62, 63, 66–68, 73–76, 80, 81, 93] were overrepresented in the included studies. The medical specialties oncology and orthopedic surgery were the most often investigated single specialties within the studies (*n* = 16 and *n* = 9, respectively) [35, 36, 38, 40, 44–46, 48, 52, 54, 56, 59, 62, 66, 71, 81–83, 85, 88, 91, 95, 96, 106], while other medical specialties (e.g., internal medicine or otolaryngology) were only represented in studies combining several medical fields (*n* = 11) [43, 49, 51, 53, 57, 58, 61, 63, 64, 72–76, 84].

**Table 1:** Overview of the characteristics of studies included in this scoping review

Qualitative studies				Quantitative studies			
First author, year	Country	Study design	Reference	First author, year	Country	Study design	References
				Allergology			
				Moscato 2014	Italy	Cross-sectional	[77]
Cardiology				Cardiology			
Frank 2018	Sweden	Focus groups	[41]	Mirmohammadi 2013	Iran	Prospective cohort	[100]
				Dermatology			
				Keegel 2007	Australia	Cross-sectional	[97]
				Skudlik 2008	Germany	Prospective cohort	[103]
				Emergency medicine			
				Rowe 2018	Canada	Prospective cohort	[99, 102]
				Hayman 2021	Canada	Cross-sectional	[69]
				Jauregui 2020	USA	Retrospective cohort	[105]
				Walker 2007	UK	Cross-sectional	[90]
				General surgery			
				Fowler 2019	Australia and New Zealand	Cross-sectional	[65]
				Grewal 2014	UK	Cross-sectional	[86]
				Gynecology and obstetrics			
				Clinch 2009	USA	Prospective cohort	[98]
				Gustavsson 2013&2016^c,e,f,g^	Sweden	Cross-sectional	[67, 68]
				Naidu 2012	UK	Cross-sectional	[78]
Hand surgery				Hand surgery			
Newington 2019	UK	Interviews	[49]	Newington 2018	UK	Cross-sectional	[79]
				Ratzon 2006	Israel	Prospective cohort	[101]
Hepatology							
Bailey 2019	USA	Interviews	[34]				
				Neurology			
				Snöljung 2017^ g^	Sweden	Cross-sectional	[80]
Oncology				Oncology			
Bains 2012	UK	Interviews	[35]	Bränström 2014^f^	Sweden	Cross-sectional	[62]
Dugan 2021	USA	Open-ended questionnaire	[40]	Braybrooke 2015	UK, France, Germany	Cross-sectional	[91]
Lamort-Bouché 2021	France	Interviews	[44]	Kirchhoff 2017	USA	Cross-sectional	[71]
MacLennan 2017	UK	Interviews	[45]	Salit 2020	USA	Cross-sectional	[88]
Main 2005	USA	Interviews	[46]	Söderman 2021	Sweden	Cross-sectional	[81]
Morrison 2015	Canada	Interviews	[48]	Takahashi 2018	Japan	Cross-sectional	[95]
Thompson 2013	Australia	Mixed-methods	[56]	Wada 2012	Japan	Cross-sectional	[83]
Tiedtke 2012	Belgium	Focus groups	[54]	Zegers 2020	the Netherlands	Cross-sectional	[96]
Orthopedic surgery				Orthopedic surgery			
Bardgett 2016	UK	Interviews	[36]	Arrelöv 2007^e^	Sweden	Cross-sectional	[59]
Coole 2019	UK	Interviews	[38]	Grevnerts 2018	Sweden	Cross-sectional	[66]
Szekeres 2018	UK	Interviews and observations	[53]	Jenny 2016	France	Retrospective cohort	[106]
Swartling 2008	Sweden	Interviews	[52]	Tsang 2020	UK	Cross-sectional	[82]
				Watson 2009	USA	Cross-sectional	[85]
				Pulmonology			
				Barber 2007	UK	Cross-sectional	[60]
				Holness 2007	Canada	Cross-sectional	[70]
Rehabilitation medicine				Rehabilitation medicine			
Holmlund 2021	Sweden	Focus groups	[42]	Michel 2018	France	Cross-sectional	[87]
Paniccia 2019	Canada	Interviews	[50]	O’Hagan 2011	Canada	Cross-sectional	[94]
				van Velzen 2020	the Netherlands	Cross-sectional	[89]
Rheumatology				Rheumatology			
Decuman 2015	Belgium	Interviews	[39]	de Croon 2005	the Netherlands	Cross-sectional	[92]
Hollick 2020	UK	Mixed-methods	[55]	Meunier 2016	France	Cross-sectional	[47]
van der Meer 2011	the Netherlands	Interviews	[108]	Zirkzee 2008	the Netherlands	Prospective cohort	[104]
Studies combining medical specialties				Studies combining medical specialties			
Bosma 2020	the Netherlands	Focus groups	[37]	Alexander 2012	UK	Cross-sectional	[57]
Kosny 2018^a^	Canada	Interviews	[43, 51]	Allen 2010	UK	Cross-sectional	[58]
				Bayliss 2020	UK	Cross-sectional	[61]
				Lindholm 2010^d,f^	Sweden	Cross-sectional	[63, 73, 74]
				Clayton 2007	UK	Cross-sectional	[64]
				Ladak 2021	Canada	Cross-sectional	[72]
				Ljungquist 2015^ g^	Sweden	Cross-sectional	[75]
				Löfgren 2007^e^	Sweden	Cross-sectional	[76]
				Nilsing 2014	Sweden	Cross-sectional	[93]
				Steenbeek 2014	the Netherlands	Prospective longitudinal	[107]
				Walters 2010	UK	Cross-sectional	[84]

*UK* United Kingdom; *USA* United States of America

^a^Kosny 2018 and Russel 2019 reported on the same data; ^b^Rowe 2018 and Gaudet 2019 reported on the same data; ^c^Gustavsson 2013 and Gustavsson 2016 reported on the same data; ^d^Lindholm 2010, Bränström 2013, and Ljungquist 2013 reported on the same data; ^e^Part of large Swedish cross-sectional study in 2004; ^f^Part of large Swedish cross-sectional study in 2008; ^g^Part of large Swedish cross-sectional study in 2012

### Synthesis of Findings

Table 2 provides an overview of the six main categories with their subcategories and the corresponding studies that provided the findings. Most qualitative studies contained information covering four to five categories. Most quantitative studies provided information on only one or two categories. Details of the extracted quantitative findings (organized per main category) can be found in Table 3. Detailed descriptions of all subcategories are provided below.

**Table 2:** Overview categories and subcategories and which studies covered them

Category and subcategory	Qualitative studies		Quantitative studies	
	*n*	References	*n*	References
1. Exploration of work and disease work-relatedness by medical specialists				
1.1 Collecting information on a patient’s occupation	1	[38]	4	[61, 82, 83, 87]
1.2 Establishing work as the cause of injury or disease	2	[43, 53]	4	[60, 70, 77, 87]
1.3 Exploring the context of work to provide advice	4	[36, 38, 45, 53]	3	[65, 66, 105]
2. Discussing work-related concerns with patients				
2.1 To discuss or not to discuss work-related concerns	10	[36–39, 41, 44, 46, 48, 55, 56]	8	[47, 56, 58, 72, 82, 87, 95, 96]
2.2 Timing of discussing work-related concerns	6	[36, 41, 44, 46, 50, 53]	–	
2.3 Initiator of the discussion about work	4	[35, 43, 44, 48]	2	[72, 82]
3. Nature of the work-related advice given to patients				
3.1 Providing general health advice	4	[35, 44, 48, 49]	4	[79, 92, 97, 98]
3.2 Patient–physician collaboration in work-related advising	5	[34–36, 45, 53]	8	[69, 71, 78, 85, 91, 94, 104, 106]
3.3 Staying at work and RTW advice for temporary conditions and post-operative periods	9	[35, 36, 38, 42, 45, 49, 52–54]	11	[64, 69, 78, 79, 83, 85, 86, 88, 91, 99, 101, 102]
3.4 Work-related advice for chronic diseases	5	[37, 38, 43, 46, 50]	5	[60, 70, 92, 97, 100]
4. Other work-related support for patients				
4.1 Scheduling care around work	2	[37, 38]	1	[83]
4.2 Tailored vocational rehabilitation	1	[36]	4	[87–89, 103]
4.3 Work-related support guided by systems for the rehabilitation of sick and injured workers	8	[41–43, 48, 49, 51–54]	19	[57–60, 62, 63, 67–69, 72–75] [76, 80, 81, 83, 84, 88, 90, 93, 107]
5. Interdisciplinary cooperation between medical specialists and other healthcare providers				
5.1 Referral to other professionals	5	[36, 44, 48, 53, 54]	10	[59, 60, 62, 67, 77, 79, 80, 83, 88, 97]
5.2 Providing work-related support in collaboration with other professionals	6	[41–44, 51, 53, 108]	6	[62, 80, 83, 87–89]
6. Medical guidelines integrating work and the use of these guidelines by medical specialists	3	[35, 48, 53]	18	[60, 64, 65, 68–70, 77–79, 81, 82, 84, 86, 88, 89, 97, 99, 102, 106]

**Table 3:** Main outcomes of quantitative studies

First author, year	Medical specialty	Main outcome	References
**Exploration of work and disease work-relatedness by medical specialists**			
Barber 2007	Pulmonology	The reported clinical approach for diagnosis in a clinical case scenario of possible occupational asthma attributed to flour dust exposure, as percentage of total number of general respiratory physicians without a specific interest in occupational lung disease (*n* = 45): –History taking: 46% asked about the relationship between work and symptoms or improvement of health when away from work –Diagnostics: 14% explored work-related trends using specific software (OASYS-2); 47% used either skin prick tests of specific IgE to flour (agent of exposure in case scenario)	[60]
Bayliss 2020	Cardiology, obstetrics, and gynecology, oncology, orthopedic surgery	Percentage of doctors asking always or most of the time about patient’s occupation: 93% of total sample (*n* = 42); 93% of cardiology (*n* = 15); 67% of obstetrics and gynecology (*n* = 9); 50% of oncology (*n* = 6); and 100% of orthopedic surgery (*n* = 12)	[61]
Holness 2007	Pulmonology, primary care	Percentage of pulmonologists (*n* = 65): –Taking history of workplace exposure: always/most of the time, 92%; sometimes, 6%; and rarely/never, 2% –Conducting investigation of patients with possible occupational asthma: always diagnose condition myself, 27%; sometimes refer to specialist, 50%; mostly refer to specialist, 23%	[70]
Moscato 2014	Allergology	From cases of occupational asthma (*n* = 80), 27.5% of patients underwent specific exposure challenge with suspected professional agent during diagnostics	[77]
Michel 2018	Rehabilitation	Percentage of rehabilitation centers offering a functional restoration program for chronic lower back pain patients reported (*n* = 56): –89% collecting occupational information at inclusion –66% collecting occupational information at the end of the program –91% collecting occupational information during an individual interview –71% collecting occupational information through a self-administered questionnaire –100% investigating the link between lower back pain and occupational activity, especially in case of work injury The content of collected information mostly concerned the current social and occupational status. Opinions about fitness for work were collected in less than half of the cases. Workplace environment was the most poorly discussed aspect	[87]
Tsang 2020	Orthopedics	Percentage of surgeons identifying whether patients are working and intending to RTW after surgery (*n* = 78): 10% of orthopedic surgeons	[82]
Wada 2012	Oncology	As reported by oncologists (*n* = 668): –I learn the type and quantity of the patient’s work: 27.8% strongly agree, 38.3% agree, 27.0% disagree, and 6.9% strongly disagree –The medical institution facilitates an interview sheet that requires a description of information on the patient’s work: 52.8% strongly agree, 7.2% agree, 3.3% disagree, and 32.6% strongly disagree	[83]
**Discussing work-related concerns with patients**			
Allen 2010	23 medical specialties, not further specified	Percentage of training-grade doctors discussing work ability in patients off sick for four weeks or less (*n* = 918): 15% weekly, 68% rarely or never	[58]
Ladak 2021	Rheumatology, dermatology, gastroenterology	Percentage of respondents reporting being asked for advice on RTW during the COVID-19 pandemic (*n* = 151): 94%	[72]
Meunier 2016	Rheumatology	–Percentage of rheumatologists reporting having a work-related discussion when asked to describe their most recent consultation with a rheumatoid arthritis patient (*n* = 153): 50%. Of this group, the frequency of discussing the following subjects was psychological problems related to work, 29%; physical problems related to work, 88%; adaptation of working conditions, 34%; impact of long-term sick leave, 26%; return to work, 10%; difficulties in going to work, 31%; and disclosure of the disease to the employer, 55% –Percentage of rheumatoid arthritis patients reporting having a work-related discussion when asked to describe their most recent consultation with a rheumatologist (*n* = 81): 52%. Of this group, the frequency of discussing the following subjects was psychological problems related to work, 24%; physical problems related to work, 88%; impact of long-term sick leave, 43%; return to work, 29%; difficulties in going to work, 21%; and disclosure of the disease to the employer, 36%	[47]
Michel 2018	Rehabilitation	Percentage of rehabilitation centers offering a functional restoration program to chronic low back pain patients reported (*n* = 56): –84% discussed obstacles for RTW –90% discussed feasible workstation arrangements	[87]
Takahashi 2018	Oncology	Percentage of patients with cancer reporting to be asked about work-related difficulties by healthcare providers (*n* = 950): 23.5%; of this group, 140 (63.3%) reported this was asked by a physician	[95]
Thompson 2013	Oncology	Oncologists’ practice (*n* = 6) of discussing strategies to integrate treatment with educational, employment, and social lives (on 5-point Likert scale from unlikely (1) to likely (5)): 1 (0.8), median (IQR)	
Tsang 2020	Orthopedics	Percentage of orthopedic surgeons providing RTW advice when a patient specifically asked (*n* = 78): 96% Percentage of surgeons reporting whether patients in work and intending to return to work after surgery receive additional advice and support during their in-patient stay or after discharge (*n* = 78): 8%	[82]
Zegers 2020	Oncology	Cancer survivors’ indication that a healthcare professional within the hospital had discussed the work-related consequences of cancer and/or its treatment with them (*n* = 3500): 992 (32%); of these conversations, 508 (53%) were with a physician or medical specialist	[96]
**Nature of work-related advice given to patients**			
Barber 2007	Pulmonology	Advice from general respiratory physicians (*n* = 45) if occupational asthma would be confirmed: –63% advised some form of reduction in exposure (personal protective equipment or alternative employment)	[60]
Braybrooke 2015	Oncology	28.6% of French (*n* = 77), 28.6% of German (*n* = 58), and 25.0% UK participants (*n* = 63) received information about RTW 35.7% of French (*n* = 77), 14.3% of German (*n* = 58), and 8.2% of UK participants (*n* = 63) received encouragement to RTW	[91]
Clayton 2007	Gynecology, orthopedic surgery, primary care, and occupational health care	Gynecologist responses for benign abdominal hysterectomy procedures (n = 11): –Advice regarding office work: 6 (55%) gynecologists advised RTW within 6 weeks, 4 (36%) within 8 weeks, and 1 (9%) within 10 weeks –Advice regarding moving and handling (M + H) duties: 4 (36%) gynecologists advised RTW between 4 and 9 weeks, 6 (55%) within 10–12 weeks, and 1 (9%) at 12 weeks No information reported on return to work form hysterectomy ‘patient information’ leaflets (*n* = 10) Consultant responses for Birmingham hip resurfacing procedures (BHR) (*n* = 13): –Advice regarding office work: 6 (46%) orthopedic surgeons advised RTW within 6 weeks –Advice regarding moving and handling (M + H) duties: 8 (62%) orthopedic surgeons advised RTW within 12 weeks –Advice regarding heavy physical work: 10 (77%) orthopedic surgeons would only occasionally advice return to heavy physical work Note: 3 orthopedic surgeons (23%) advocated RTW within other periods of time, including a return to desk work within 2.5 weeks and longer than 12 weeks if other joint problems were present Specific return to work times were stated in 4 BHR ‘patient information’ leaflets (*n* = 11). Three advised RTW within 6–8 weeks of surgery Without stipulating any limitations on the type of work other than general activities to be avoided such as squatting, twisting, or prolonged standing. One advised return to office work after 6 weeks but if work involved prolonged standing, the patient should refrain from work for 3 months	[64]
Clinch 2009	Obstetrics	There was substantial variation in the content of women’s RTW discussions with healthcare providers: –Content of RTW discussions with Prenatal Health Care Provider (*n* = 131) concerned: 19.5% maternal health (i.e., physical or mental); 10.6% infant’s health or development; 23.6% both mother and infant’s health; and 46.3% other –Content of RTW discussions with Infant’s Health Care Provider concerned (*n* = 125): 8.4% maternal health (i.e., physical or mental); 33.6% infant’s health or development; 22.7% both mother and infant’s health; and 35.3% other	[98]
de Croon 2005	Rheumatology	Advice from rheumatologists (*n* = 78): –8% gave organizational advice, including advice such as adjustment of tasks or work schedules –3% gave technical advice, including advice such the application of assistive devices (e.g., application of a knee table to write) –13% gave personal advice including personal adjustments to maintain work ability, which were not specific (e.g., ‘do not force yourself’) Receiving advice on how to maintain work ability from other medical specialists: the surgeon (5%), the rehabilitation physician (4%), and the orthomanual physician (1%)	[92]
Fowler 2019	Colorectal surgery	The reported surgeon’s choice of treatment in a clinical scenario describing a patient with grade IV hemorrhoids with preference of prompt RTW after surgery, percentage of surgeons (*n* = 82): –Stapled hemorrhoidectomy or Doppler-guided hemorrhoidectomy, in concordance with guideline recommendations, 58% –Conventional excisional hemorrhoidectomy; despite the reportedly longer return to normal activities post-procedure, 36%	[65]
Grevnerts 2018	Orthopedic surgery and physical therapy	Percentage of orthopedic surgeons (*n* = 98): –Who would advise surgery when patient’s occupation is physically demanding for the knee and requires knee stability: 96% –Reporting knee instability in working situations as the most important factor for advising surgery: 2% –Reporting work in combination with other factors as the most important for advising surgery: 6%	[66]
Grewal 2014	General surgery	Acute trusts providing inguinal hernia repair (*n* = 128) reported a wide variation in the advice given to patients by their operating surgeons regarding the time to return to work: –Open inguinal hernia repair: From 1 week to 4–6 weeks for office work; from 2 weeks to 6–12 weeks for manual labor –Laparoscopic inguinal hernia repair: From 1 week to 2–4 weeks for office work; from 2 weeks to 2–4 weeks for manual labor	[86]
Hayman 2021	Emergency health care	Advice to remain home for minor illness (*n* = 182): most physicians answered that they advise patients to remain at home for minor illnesses; however, the duration of exclusion from work varied; for influenza-like illness, the median was 4 days, 6.5% answered until fever resolves; for upper respiratory tract infection, the median was 2 days, 7.1% answered until fever resolves; and for gastroenteritis, the median was 2 days, 9.0% answered until fever resolves Percentage of respondents who believed that a patient is capable of determining when to return to work (*n* = 182): most of the time 82.8%, sometimes 17.2%, and rarely 0%	[69]
Holness 2007	Pulmonology and primary care	Percentage of pulmonologists (*n* = 65) advising patients with suspected occupational asthma to leave work until diagnosis is confirmed: 5% always and 27% most of the time (other 68% was not reported by the authors)	[70]
Jauregui 2020	Emergency medicine	Acute low back pain that was the result of a work-related injury was shown to have an inverse relationship with the likelihood that an opioid was prescribed by emergency physicians. Patients (*n* = 162) whose injury was work related were less likely to receive an opioid prescription (*p* = 0.027, 95% CI)	[105]
Jenny 2016	Orthopedic surgery	From the study protocol, progressive RTW was allowed according to patient’s decision. No restriction was imposed by the physician at any follow-up time or for any work activity	[106]
Keegel 2007	Dermatology	Advice from dermatologist in cases of workers suspected of occupational contact dermatitis (*n* = 44): –Percentage of dermatologists providing general advice on medical treatment and preventive measures: oral steroids/immunosuppressive agent, 54.5%; corticosteroid creams, 9.1%; corticosteroid ointment, 43.2%; topical corticosteroid not specified, 34.1%; moisturizing cream, 9.1%; moisturizer not specified, 18.2%; using gloves, 38.6%; using soap substitute/avoiding soap, 0%; antibiotics, 22.7%; and antihistamines, 0% –Percentage of dermatologists providing general advice for further diagnostics: fungal scrapings or radioallergosorbent test, 4.5% –Percentage of dermatologists providing specific work advice: avoidance/modify duties, 20.5%; change of job, 4.5% –Referring practices: treat patients independently, 2.3%; refer on initial presentation to tertiary occupational dermatology clinic, 81.8%; and no information, 15.9%	[97]
Kirchhoff 2017	Oncology	23.9% of oncologists (*n* = 91) reported that their adolescent and young adult (AYA) cancer patients needed assistance with employment support; this support is always or often unmet for AYAs according to 16.9% of oncologists	[71]
Mirmohammadi 2013	Cardiology	From patients not returning to work after first myocardial infarction (*n* = 40), 7.5% gave physician’s order as the reason and 22.5% gave physician’s recommendation as the reason	[100]
Naidu 2012	Gynecology and obstetrics	Advice given by obstetrician and gynecologists (*n* = 472) regarding time in days before return to convalescence activities (median, 25–75 percentiles): diagnostic hysteroscopy, 2 days (1–2); operative hysteroscopy, 7 days (2–7); diagnostic laparoscopy, 7 days (2–7); operative laparoscopy, 10 days (5–14); abdominal surgery, 42 days (42–56); and vaginal surgery, 42 days (28–56) In addition, conditional advice for RTW as an open-ended question response (number of respondents), when comfortable (n = 14) and depends on work (*n* = 12)	[78]
Newington 2018	Surgery and occupational and physical therapy	Advice for patients undergoing carpal tunnel release surgery given by surgeons (*n* = 173) regarding time in days before return to work (median, IQR): desk-based duties (e.g., keyboard, mouse, writing, telephone), 7 days (2–14); repetitive light manual duties (e.g., driving, delivery, stacking), 14 days (14–28); and heavy manual duties (e.g., construction), 30 days (21–42) Additional recommendations from open-ended question (% of surgeons): advice to limit function to clean and/or dry activities until the wound was healed (31%); avoiding activities that might aggravate the surgical site, such as heavy gripping or weight-bearing through the hand during 2–6 weeks (34%); advice to resume driving ranged from the day of surgery to 6 weeks postoperatively (30%); and advice was dependent on the patient’s individual circumstances (42%)	[79]
O'Hagan 2011	Cardiac rehabilitation	Perceived physician support to RTW for patients (*n* = 214) on 5-point Likert scale (1–5) (SD): working patients: 4.21 (0.74) and disabled patients: 3.71 (1.10); *p* = 0.002	[94]
Ratzon 2006	Surgery	Surgeon’s recommendation on RTW after carpal tunnel release surgery (*n* = 50): 1–36 days (median 21; IQR 14–30) Surgeon’s recommendations were the strongest predictor of delayed RTW (> 21 days), this was not correlated with objective findings	[101]
Rowe 2018^a^	Emergency medicine	Frequency of recommendation to stay home from work following a concussion after emergency department discharge by emergency physicians (*n* = 197): 69% Patients provided with the ‘Electronic Clinical Practice Guidelines’ handout (*n* = 119) were more frequently recommended to avoid work (OR 1.95, 95% CI 1.10–3.48) than those who did not receive the handout (*n* = 131) Patients injured at work (*n* = 28) were more likely to receive advice to miss work (OR 3.1; 95% CI 1.0–9.2) than those not injured at work (*n* = 169)	[99, 102]
Salit 2020	Oncology	Advice on RTW of transplantation centers performing > 50 total Hematopoietic Cell Transplantations (HCT) per year based on their local guidelines (*n* = 45): –Autologous HCT: 70%, 1–3 months post-HCT, 70%; 4–6 months post-HCT, 30% –Allogeneic HCT: 4–6 months post-HCT, 20%; 6–12 months post-HCT, 60%; until cessation of immune suppression, 11%; and no specific guideline, 5% –Job types that require longer absence from work: interacting with sick people, 70%; interacting with children, 65%; interacting with people, 40%; interacting with animals, 50%; jobs with significant physical activity, 35%; no specific guideline, 25%; and jobs with significant travel, 10%	[88]
Wada 2012	Oncology	As reported by oncologists (*n* = 668): –I explain the impact of treatment on work: 53.0% strongly agree, 40.0% agree, 6.1% disagree, 0.9% strongly disagree	[83]
Watson 2009	Orthopedic surgery	Probability (%, CI) of surgeons (*n* = 125) recommending to RTW with radiographic union in different scenarios at two points in time after injury: Motivated athlete with pain: 24wks, 85% (78–90); 52wks 88% (82–92) | Motivated athlete no pain: 24wks, 100% (99–100); 52wks 100% (99–100) | Unmotivated office worker with pain: 24wks, 98% (97–99); 52wks 99% (97–99) | Unmotivated office worker no pain: 24wks, 100% (99–100); 52wks 100% (99–100) | Unmotivated laborer with pain: 24wks, 85% (78–90); 52wks 89% (84–93) | Unmotivated laborer no pain: 24wks, 100% (99–100); 52wks 100% (99–100) | Motivated laborer with pain: 24wks, 96% (93–97); 52wks 96% (94–98) |Motivated laborer no pain: 24wks, 100% (99–100); 52wks 100% (99–100) Probability (%, CI) of surgeons (n = 125) recommending to RTW with radiographic *non*union in different scenarios at three points in time after injury: Motivated athlete with pain: 6wks, 7% (4–10); 24wks, 15% (10–22); 52wks 18% (41–58) | Motivated athlete no pain: 6wks, 24% (17–23); 24wks, 44% (36–53); 52wks 50% (82–92) | Unmotivated office worker with pain: 6wks, 62% (53–70); 24wks, 67% (57–75); 52wks 68% (59–76) | Unmotivated office worker no pain: 6wks, 94% (88–97); 24wks, 95% (90–98); 52wks 96% (90–98) | Unmotivated laborer with pain: 6wks, 4% (2–6); 24wks, 15% (10–22); 52wks 20% (15–28)| Unmotivated laborer no pain: 6wks, 13% (8–21); 24wks, 43% (34–52); 52wks 52% (43–60) | Motivated laborer with pain: 6wks, 25% (18–33); 24wks, 40% (32–49); 52wks 45% (36–53) | Motivated laborer no pain: 6wks, 59% (49–67); 24wks, 74% (66–81); and 52wks 77% (70–84)	[85]
Zirkzee 2008	Rheumatology	Usage of professional guidance from rheumatologist with respect to arthritis-related problems at work for patients with early arthritis (*n* = 69): at study entry, 54%; at 12-month follow-up, 30%	[104]
**Other work-related support for patients**			
Alexander 2012	All medical specialties	Frequency of writing sick note certificate (*n* = 40): 15% every week, 35% every month, and 45% rarely	[57]
Allen 2010	23 medical specialties, not further specified	Frequency of writing sickness certificates (*n* = 918): 14% monthly or more, 85% rarely or never	[58]
Arrelöv 2007	Orthopedic surgery and primary care	Frequency for orthopedic surgeons (*n* = 149): –Having consultations that include considerations of sickness certification: 97.3% weekly, 2.7% monthly, 0.0% yearly, 0.0% never –Issuing sickness certificates without personal appointment: 36.5% weekly, 23.0% monthly, 16.2% yearly, 24.3% never –Having contact with social insurance office about matters concerning sickness certification: 10.7% weekly, 28.9% monthly, 42.3% yearly, 18.1% never	[59]
Barber 2007	Pulmonology	28% of general respiratory physicians (*n* = 45) specifically mentioned either providing compensation advice or writing a referral for the opinion of a specialist in occupational asthma	[60]
Lindholm 2010^b^	All medical specialties	Physicians having sickness certification consultations at least a few times a year (n (% of total group)) and the frequencies during a week of these consultations (% of subgroup): –Overall: 14,210, 63.6%; 6% > 20 times a week, 34% 6–20 times a week, 42% 1–5 times a week, 12% about once a month, 5% a few times a year –Internal medicine: 1963, 97.4%; 2.4% > 20 times a week, 19.1% 6–20 times a week, 50.0% 1–5 times a week, 28.5% < once a week –Surgery: 1376, 88.1%; 4.6% > 20 times a week, 33.4% 6–20 times a week, 48.1% 1–5 times a week, 13.8% < once a week –Gynecology: 930, 86.9%; 1.8% > 20 times a week, 29.6% 6–20 times a week, 50.6% 1–5 times a week, 18.0% < once a week –Orthopedics: 898, 95.6%; 19.2% > 20 times a week, 59.3% 6–20 times a week, 18.5% 1–5 times a week, 3.1% < once a week –Ophthalmology: 358, 73.2%; 0.0% > 20 times a week, 3.8% 6–20 times a week, 21.8% 1–5 times a week, 74.4% < once a week –Ear, nose, and throat: 445, 91.6%; 0.9% > 20 times a week, 21.0% 6–20 times a week, 60.0% 1–5 times a week, 18.0% < once a week –Infectious diseases: 331, 97.0%; 1.6% > 20 times a week, 18.6% 6–20 times a week, 60.7% 1–5 times a week, 19.2% < once a week –Oncology: 334, 96.0%; 20.2% > 20 times a week, 50.9% 6–20 times a week, 23.9% 1–5 times a week, 5.0% < once a week –Dermatology: 208, 78.5%; 0.0% > 20 times a week, 3.5% 6–20 times a week, 22.0% 1–5 times a week, 74.5% < once a week –Neurology: 244, 94.2%; 2.6% > 20 times a week, 42.7% 6–20 times a week, 47.8% 1–5 times a week, 6.9% < once a week –Rheumatology: 191, 99.0%; 3.3% > 20 times a week, 45.6% 6–20 times a week, 45.6 1–5 times a week, 10.7% < once a week –Rehabilitation: 177, 93.2%; 17.9% > 20 times a week, 45.2% 6–20 times a week, 26.2% 1–5 times a week, 10.7% < once a week –Pain management: 85, 75.9%; 17.8% > 20 times a week, 56.2% 6–20 times a week, 20.5% 1–5 times a week, 5.5% < once a week	[63, 73, 74]
Bränström 2014	Oncology	Frequency of sickness certification consultations by physicians working mainly at oncology clinics (*n* = 348): 67.2% > 6 times a week, 23.6% 1–5 times a week, 9.2% < sometimes each month	[62]
Gustavsson 2013&2016^c^	Gynecology/obstetrics	Frequency of sickness certification consultations by physician working in gynecology/obstetrics: –In 2004 (*n* = 315): 33.7% > 6 times a week, 37.5% 1–5 times a week, 18.4% a few time per month or year, 10.2% never of almost never –In 2008 (*n* = 1037): 27.4% > 6 times a week, 43.9% 1–5 times a week, 16.9% a few time per month or year, 11.3% never of almost never –In 2012 (*n* = 992): 18.8% > 6 times a week, 48.0% 1–5 times a week, 21.6% a few time per month or year, 11.5% never of almost never	[67, 68]
Hayman 2021	Emergency health care	Percentage of physicians providing sick notes (*n* = 182): 76.4% at least once a day, of which 4.2% 5 or more a day	[69]
Ladak 2021	Rheumatology, dermatology, gastroenterology	Percentage of physician providing a note for delayed RTW or modified duties (*n* = 151): 25% (range 0–100%; 10–77.5, interquartile range 67.5) Percentage of physicians providing a delayed RTW/modified duties note in seven clinical scenarios (*n* = 151): 1. DMARDs only, 5.4% (3.1% was unsure); 2. DMARDs and poly-pharma, 8.0% (9.6% was unsure); 3. DMARDs, poly-pharma, biologics and risk during commute, 21.0% (5.6% was unsure); 4. DMARDs, poly-pharma and steroids, 32.3% (8.9% was unsure); 5. Poly-pharma, biologics, steroids, vulnerable individuals at home, risk at work, comorbidity, and over age 60, 57.3% (15.3% was unsure); 6. DMARDs, poly-pharma, biologics, risk during commute, vulnerable individuals at home and risk at work, 59.7% (14.5% was unsure); 7. DMARDs, poly-pharma, biologics, risk during commute, vulnerable individuals at home, risk at work, and comorbidity, 74.2% (8.9% was unsure)	[72]
Ljungquist 2015	All medical specialties	Frequency of sickness certification consultations: –Orthopedics (*n* = 850): 71% > 5 times per week, 26% 1–5 times per week –Rehabilitation (*n* = 168): 59% > 5 times per week, 33% 1–5 times per week –Pain management (*n* = 73): 68% > 5 times per week, 23% 1–5 times per week –Oncology (*n* = 322): 60% > 5 times per week, 31% 1–5 times per week –Rheumatology (*n* = 182): 27% > 5 times per week, 59% 1–5 times per week –Neurology (*n* = 232): 35% > 5 times per week, 51% 1–5 times per week –Surgery (*n* = 1307): 29% > 5 times per week, 50% 1–5 times per week –Gynecology/obstetrics (*n* = 878): 21% > 5 times per week, 54% 1–5 times per week –Infectious diseases (*n* = 318): 15% > 5 times per week, 58% 1–5 times per week –Internal medicine (*n* = 1875): 14% > 5 times per week, 48% 1–5 times per week –Dermatology (*n* = 200): 2% > 5 times per week, 7% 1–5 times per week	[75]
Löfgren 2007	All medical specialties	Percentage of respondents having sickness certification consultations at least a few times a year: All specializations (*n* = 5455), 74%; internal medicine (*n* = 396), 92%; surgery (*n* = 218), 93%; gynecology / obstetrics (*n* = 215), 90%; orthopedics (n = 200), 95%; oncology (*n* = 108), 97%; rehabilitation care (*n* = 75), 77 Frequency of sickness certification consultations for physicians having these consultations at least a few times a year (*n* = 4019): 9.4% > 20 times a week, 41.0% 6–20 times a week, 34.3% 1–5 times a week, 8.5% about once a month, 5.8% a few times a year	[76]
Michel 2018	Rehabilitation	Percentage of rehabilitation centers offering a functional restoration program for chronic lower back pain patients reported (*n* = 56): –29% offered a specific work rehabilitation program –77% involved a rehabilitation physicians in RTW management –100% investigated the patient’s work plan –18% adapted their programs regarding workplace information Main actions to improve a patient’s transition from the healthcare setting to the workplace were: to refer the patient to the occupational physician for a pre-RTW medical examination (94%), to contact the occupational physician (89%), to request the recognition of disabled worker status (66%), and to refer the patient to a social worker (62%)	[87]
Nilsing 2014	Primary health care, occupational health services, private clinics, and hospital care	Prescription of part-time sick leave by physicians at hospitals (*n* = 245): 6% Prescribed intervention of physicians at hospitals in context of sickness certificate (*n* = 245): 26% no intervention, 39% medical intervention, 27% early rehabilitation (< 28 days after starting current sick leave period), and 7% late rehabilitation (> 28 days after starting current sick leave period)	[93]
Salit 2020	Oncology	Percentage of transplantation centers performing > 50 total hematopoietic cell transplantations (HCT) per year (*n* = 45) that have a RTW program: 36% Composition of the RTW programs: –Programs entailed paperwork, 81%; formal group program, 19%; one-on-one counseling, 100%; videos, 13%; and vocational rehabilitation, 25% –Program is run/maintained by physician, 38%; advanced practice provider, 19%; nurse, 6%; social worker, 81%; psychologist, 25%; rehabilitation specialist, 25%; and financial counselor, 6% –Number of sessions: one-time offering, 19%; three sessions per year, 6%; unlimited depending on need, 13%; and not applicable, 62% –Start of the program: when the patient arrives for transplantation, 6%; as the patient is preparing to leave the center, 19%; at the one-year follow-up visit, 6%; and when the patient is ready to participate, 38% Sickness certification: 85% of all centers completed disability paperwork at > 1 year post-HCT for patients who are actively under their care –Criteria for considering a patient disabled include: the patient reports an inability to work, 38%; the patient has persistent or relapsed disease, 78%; the patient is still on immune suppression drugs, 38%; the patient has ongoing health complications and/or chronic graft versus host disease, 89%; and to maintain the patient’s health insurance, 4%	[88]
Skudlik 2008	Dermatology	Tertiary individual prevention (TIP) is a specific tertiary interdisciplinary in-patient prevention measure program developed for severe cases of occupational skin disease (OSD), in which outpatient prevention measures are not successful. This program was established in 1994 as part of regular health care. In total, 1486 patients took part in TIP between 1994 and 2003 TIP consists of several components: –In-patient phase of 2–3 weeks in which dermatological and health educational measures are central. Dermatological therapy is carried out by dermatologists specialized in occupational dermatology. The health educational measures are organized interdisciplinary and run parallel –Outpatient no exposure phase of 3 weeks in which dermatological therapy is continued by the local dermatologist –Each patient will stay out of work for usually 6 weeks to allow full recovery. At the end of this period the patient returns to his workplace under further dermatological care and with the provision of optimized skin protective measures as well as optimized work organization measures. The medical course is continuously documented –In parallel, an intensified health educational intervention is carried out via group seminars and individual consultations with regard to the required, occupation-specific skin protection in each individual case (i.e., skin protective measures are tested with the support of occupational therapists in models of workplace environments; health-psychological interventions are offered; consultations with specialized job consultants of the statutory employers’ liability insurances are provided regarding insurance/legal questions or to initiate changes in the work organization and work environment)	[103]
Snöljung 2017	Neurology	Frequency of sickness certification consultations by neurologists (*n* = 265): 32.8% > 5 times a week, 48.7% 1–5 times a week, 10.6% about once month, 2.6% a few times a year, and 5.3% never / no answer Frequency of issuing sickness certificates without seeing them (e.g., by telephone) by neurologists (*n* = 265): 23.2% at least once a week, 64.0% about once a month or a few times per year, and 12.8% never or almost never	[80]
Söderman 2021	Oncology	Frequency of sickness certification consultations (*n* = 342): 36.6% > 10 times/week; 24,6% 6–10 times/week; 31.0% 1–5 times/week; 5.9% sometimes/monthly; and 2.1% sometimes/yearly	[81]
Steenbeek 2014	Not specified	Medical specialist playing a role in preventing sickness absence for workers who reported no sickness absence at all during the study period and who reported that a health care provider had played a role in preventing sickness absence (*n* = 86): 3.5%	[107]
van Velzen 2020	Rehabilitation	Institutes providing vocational rehabilitation services (*n* = 55): 34 (62%) –Timing of vocational rehabilitation (*n* = 34): during outpatient rehabilitation: 68%; during in-patient and outpatient rehabilitation: 29%; and during in-patient and outpatient rehabilitation and after termination regular rehabilitation treatment: 3% –Assessment of the gap between the patients’ abilities and work (*n* = 34): making an overview of job requirements: 97%; making an overview of the patient’s abilities: 82%; and comparing job requirements and patient’s abilities: 97% –Vocational rehabilitation goal setting, work training, and work samples (*n* = 34): setting goals for vocational rehabilitation: 88%; training focusing on working skills: 82%; and simulated work situations as part of the training: 71% –job coaches (*n* = 34): Directing patients to job coaches after vocational rehabilitation: 74%	[89]
Wada 2012	Oncology	As reported by oncologists (n = 668): –I give consideration to minimizing the patient’s absence from work: 38.2% strongly agree, 44.9% agree, 13.8% disagree, 3.0% strongly disagree –I write the prospects for treatment and necessary considerations in the medical certificate to be submitted to the company: 25.7% strongly agree, 44.8% agree, 23.8% disagree, 5.6% strongly disagree –The medical institution facilitates patients seeing the oncologist at the appointment time: 18.9% strongly agree, 40.0% agree, 24.9% disagree, and 15.6% strongly disagree –The medical institution facilitates chemotherapy being adjusted according to work-related matters: 9.5% strongly agree, 32.4% agree, 32.7% disagree, and 22.9% strongly disagree –The medical institution facilitates radiation therapy scheduling being adjusted according to work-related matters: 7.3% strongly agree, 20.7% agree, 31.3% disagree, and 25.0% strongly disagree	[83]
Walker 2007	Emergency care	Frequency of issuing sick notes: Scottish accident and emergency departments (*n* = 25), 16%; Scottish fracture clinics (*n* = 25), 32%; English accident and emergency departments (*n* = 25), 20%; and English fracture clinics (*n* = 25), 48% Local policies on sick listing: –Give sick notes: Scottish accident and emergency departments (*n* = 25), 16%; English accident and emergency departments (*n* = 25), 20% –Not give sick notes: Scottish accident and emergency departments (*n* = 25), 12%; English accident and emergency departments (*n* = 25), 16% –No clear policy but ‘‘just don’t give them’’: Scotland accident and emergency departments (*n* = 25), 72%; England accident and emergency departments (*n* = 25), 64% –See the general practitioner if the patient required a sick note: Scottish accident and emergency departments (*n* = 25), 68%; English accident and emergency departments (*n* = 25), 52%	[90]
Walters 2010	General surgery, internal medicine, emergency medicine, general practice, orthopedics	Before issuing a sickness certification, registered doctors in specialty training (*n* = 51) ask about patient’s occupation, 96%; job type, 80%; and any adjustments that could be made to enable a patient to return to work, 50%	[84]
**Interdisciplinary cooperation between medical specialists and other healthcare providers**			
Arrelöv 2007	Orthopedic surgery and primary care	Frequency of orthopedic surgeons (*n* = 149) making a referral to occupational health: weekly 10.0%, monthly 30.9%, yearly 32.2%, and never 26.8%	[59]
Barber 2007	Pulmonology	Advice from general respiratory physicians (*n* = 45) if occupational asthma is confirmed: –14% referred for advice with a specialist in occupational asthma –23% advised patients to discuss the issues at work with the employer, occupational health provider, trade union representative, or the Health and Safety Executive	[60]
Bränström 2014	Oncology	In relation to sickness certification, frequency of physicians working mainly at oncology clinics (*n* = 348) who: –Collaborate or refer patients to physical or occupational therapists: 5.4% at least once a week, 10.9% ~ once a month, and 83.7% a few times a year or less –Collaborate or refer patients to counselors and/or psychologists: 4.9% at least once a week, 13.8% ~ once a month, and 81.4% a few times a year or less –Confer with other physicians: 2.3% at least once a week, 18.3% ~ once a month, and 79.4% a few times a year or less –Referring/sending patients to occupational health services: 0.3% at least once a week, 5.0% ~ once a month, and 94.7% a few times a year or less –Participate in a healthcare team in coordination meetings with social insurance and/or employers: 0.6% at least once a week, 2.3% ~ once a month, and 97.1% a few times a year or less –Contact employers by care team: 0.3% at least once a week, 0.3% ~ once a month, and 99.4% a few times a year or less –Contact social services: 0.0% at least once a week, 1.0% ~ once a month, and 99.0% a few times a year or less –Contact employment offices: 0.0% at least once a week, 1.0% ~ once a month, and 99.0% a few times a year or less	[62]
Gustavsson 2013&2016^c^	Gynecology/obstetrics	In relation to sickness certification, frequency of referring/sending patients to occupational health services by physician working in gynecology/obstetrics: –In 2004 (*n* = 315): 0.4% at least once a week, 23.2% a few times per month or year, and 75.4% never or almost never –In 2008 (*n* = 1037): 0.9% at least once a week, 16.1% a few times per month or year, and 79.8% never or almost never	[67, 68]
Keegel 2007	Dermatology	Referring practices of dermatologists in cases of workers suspected of occupational contact dermatitis (*n* = 44): no referral, treat patients independently, 2.3%; refer on initial presentation to tertiary occupational dermatology clinic, 81.8%; and no information, 15.9%	[97]
Michel 2018	Rehabilitation	Frequency of rehabilitation centers offering a functional restoration program (FRP) to chronic lower back pain patients reported (*n* = 56): –Disciplines involved in FRP teams (percentage of people from that discipline handling questions relative to work): rehabilitation physicians 51 (84%); rheumatologists 14 (79%); physiotherapists 54 (7%); APA teachers 46 (0%); psychologists 51 (16%); psychiatrists 16 (6%); occupational therapists 56 (55%); ergonomists 11 (91%); social workers 50 (82%); occupational physicians 8 (100%); and others 21 (38%) –Information sharing between FRP teams and Occupational Health Physician (OP) was systematic or frequent in 31 centers, rare or occasional in 20 centers, and never took place in 4 centers. These exchanges were perceived as difficult by 34 centers, mostly because of the OP’s lack of availability or for legal reasons –Information sharing was mostly undertaken through the patient (72%) or OP-addressed (57%) correspondence –FRP team composition seemed to influence the information sharing. There was a near significant relation between the frequency and the facility of information sharing and the presence of an ergonomist or of an OP in the FRP team –Half of the centers considered the information content to be insufficient because of the lack of workplace information details	[87]
Moscato 2014	Allergists	From cases of occupational asthma (*n* = 80), two-thirds were referred to the National Workers Compensation Authority (INAIL) for an occupational disease	[77]
Newington 2018	Surgery and occupational and physical therapy	Additional recommendations of surgeons from open-ended questions (*n* = 173) (% of surgeons): Involve the patients’ employers in the RTW decision-making (4%)	[79]
Salit 2020	Oncology	18% of transplantation centers performing > 50 total Hematopoietic Cell Transplantations (HCT) per year (*n* = 45) had someone (physician, advanced practice provider, nurse, social worker, or assigned liaison) contacting the patient’s employer once the patient was discharged from the transplantation team back to their primary oncologist Means of making contact: by phone, 55%; by email, 9%; and by letter, 36% Correspondence may include patient discharge summary, 19%; handout with precautions for immunocompromised patients, 19%; discussion regarding expectations for RTW, 38%; and comprehensive survivorship care plan, 13%	[88]
Snöljung 2017	Neurology	In relation to sickness certification, frequency of neurologists (*n* = 265) who –Participated independently or in a healthcare team in coordination meeting with social insurance and/or employer: 2.8% at least once a week, 47.6% about once a month or a few times per year, and 49.6% never or almost never –Independently or in a healthcare team had contact with employers in ways other than via the coordination meetings: 0.0% at least once a week, 33.1% about once a month or a few times per year, and 66.9% never or almost never –Collaborated with or referred patients to a counselor/psychologist: 10.4% at least once a week, 63.9% about once a month or a few times per year, and 25.7% never or almost never –Collaborated with or refer patients to physical or occupational therapists: 15.6% at least once a week, 66.8% about once a month or a few times per year, and 17.6% never or almost never –Conferred with other physicians: 3.2% at least once a week, 63.2% about once a month or a few times per year, and 33.6% never or almost never –Referred patients to occupational health services: 0.0% at least once a week, 55.6% about once a month or a few times per year, and 44.4% never or almost never –Had contact with social services: 0.0% at least once a week, 17.2% about once a month or a few times per year, and 82.8% never or almost never –Had contact with employment offices: 0.4% at least once a week, 53.6% about once a month or a few times per year, and 46.0% never or almost never	[80]
van Velzen 2020	Rehabilitation	From the institutes providing vocational rehabilitation services: –Disciplines involved in vocational rehabilitation (*n* = 34): rehabilitation physicians, 88%; occupational therapists, 94%; (neuro)psychologists, 82%; social workers, 91%; physiotherapists, 50%; speech therapists, 59%; nurses, 3%; and other, 29% –Involvement of external partners and transfer of information to external partners (*n* = 34): employer, 77%; occupational physician, 88%; work colleague of the patient, 38%; and other, 29% –Professionals that coordinate the vocational rehabilitation process (*n* = 34): vocational rehabilitation specialist, 29%; occupational therapist, 32%; neuropsychologist or psychologist, 6%; rehabilitation physician, 44%; social worker, 35%; physiotherapist, 3%; labor advisor, 3%; vocational rehabilitation team, 9%; and (standard) rehabilitation team, 3%, depending on the individual situation of the patient but most of the time occupational therapist, psychologist, or social worker, 3%	[89]
Wada 2012	Oncology	As reported by oncologists (*n* = 668): –I consider the involvement of a nurse in work-related counseling to be important: 34.6% strongly agree, 42.1% agree, 15.1% disagree, and 3.9% strongly disagree –I consider the involvement of a medical social worker in work-related counseling to be important: 65.0% strongly agree, 30.2% agree, 3.1% disagree, and 0.7% strongly disagree –I advise the patient to tell their supervisor about the prospects for treatment and ask for understanding: 18.1% strongly agree, 35.5% agree, 33.1% disagree, and 13.0% strongly disagree –The medical institution facilitates there being a nurse involved in work-related counseling: 8.6% strongly agree, 20.2% agree, 29.3% disagree, and 35.7% strongly disagree –The medical institution facilitates there being a medical social worker involved in work-related counseling: 25.5% strongly agree, 36.7% agree, 18.1% disagree, and 14.8% strongly disagree	[83]
**Medical guidelines integrating work and their use by medical specialists**			
Barber 2007	Pulmonology	National guidelines for the management of occupational asthma for the British Thoracic Society includes topics on diagnostics and advice Guideline recommendation on diagnostics compared to actions of general respiratory physicians (*n* = 45): –Recommends accurate history taking, in particular asking whether symptoms improve away from work and on holiday: mentioned by 46% –Does not mention diagnostic imaging: 100% would do chest x-ray and 21% CT scan to exclude other conditions –Recommends at least four peak flow measurements a day should be recorded: majority fulfilled these criteria; however, the frequency varied –Recommends immunological testing, using specific IgE measurements: 47% used either skin prick tests of specific IgE –Does not mention specific inhalation challenges; also not mentioned as diagnostic approach by physicians Guideline recommended advice compared to actions of general respiratory physicians (*n* = 45): –advice that workers diagnosed as having occupational asthma should be removed from exposure, and that relocation should occur within 12 months of the first work-related asthma symptoms, premature advice to leave the occupation is inadvisable; 63% advised some form of reduction in exposure (personal protective equipment or alternative employment)	[60]
Clayton 2007	Gynecology, orthopedic surgery, primary care, and occupational health care	Existing guideline: Department of Work and Pensions (DWP) evidence-based guidance No gynecologists (*n* = 11) or orthopedic surgeons (*n* = 13) were aware of this guideline	[64]
Fowler 2019	Colorectal surgery	Several clinical practice guidelines for the management of hemorrhoids exist with information on healing time for surgical procedures, which are of influence for return to work	[65]
Gustavsson 2013&2016^c^	Gynecology/obstetrics	Frequency of use of national sickness certification guidelines by physician working in gynecology/obstetrics: –In 2008 (*n* = 1037): every week, 8.7%, never, 52.5% (other 61.2% was not reported by the authors) –In 2012 (*n* = 992): every week, 26.7%; never, 26.4% (other 53.1% was not reported by the authors)	[67, 68]
Grewal 2014	General surgery	Recommendations to surgeons regarding post-operative care after inguinal hernia repair are available from the Royal College of Surgeons website and from the European Hernia Society guidelines. Return to work is classified into 1–2 weeks for light work, 2–3 weeks for minimal lifting, and 6 weeks for heavy labor-intensive work. The recommendations given were found to be inconsistent with these guidelines	[86]
Hayman 2021	Emergency health care	Percentage of practice environments with sick note policy (*n* = 182): –No policy, 75.1%; unsure about policy, 10.7%; and charge patients for sick note, 13%	[69]
Holness 2007	Pulmonology and primary care	No guidelines exist. Frequency of sources of information about occupational lung disease that are used by pulmonologists (*n* = 65): continuing medical education and conferences, 43%; journal articles, 58%; consultation reports from specialists, 25%; newsletters from professional organizations, 9%; information booklets from government or professional organizations, 2%; and websites, 5%	[70]
Jenny 2016	Orthopedic surgery	No consensus on RTW exists after anterior cruciate ligament reconstruction	[106]
Keegel 2007	Dermatology	Best practice guidelines for contact dermatitis and occupational contact dermatitis (OCD) exist. In no case of OCD (*n* = 44) did the dermatologists report recommending all 3 of the best practice treatment recommendations (i.e., use a soap substitute, moisturizer, and topical corticosteroid)	[97]
Moscato 2014	Allergists	Two guidelines exist (Global Strategy for Asthma Management and Prevention (GINA) and guidelines in allergic rhinitis (ARIA-GA2LEN)), but none include information on work support	[77]
Naidu 2012	Gynecology and obstetrics	Royal College of Obstetricians and Gynecologists advice on RTW: operative laparoscopy, 2–5 weeks; diagnostic laparoscopy, 1 week; operative laparoscopy, 2–3 weeks; abdominal surgery, 6–8 weeks; vaginal surgery (hysterectomy), 4–6 weeks; and vaginal surgery (prolapse), 3–4 weeks	[78]
Newington 2018	Surgery and occupational and physical therapy	Royal College of Surgeons’ patient guidance document (2014) recommends for RTW: desk-based duties, 7 days; light manual duties, 15 days; and heavy manual duties, 42 days	[79]
Rowe 2018^a^	Emergency health care	No RTW guideline present	[99, 102]
Salit 2020	Oncology	46% of transplantations centers performing > 50 total Hematopoietic Cell Transplantations (HCT) per year (*n* = 45) had local RTW guidelines: –Mechanisms by which guidelines are provided to the patient: handout, 65%; physician/advanced practice provider visit, 80%; nurse visit, 80%; social worker visit, 45%; group class, 10% –Centers that did not have guidelines commented that they determined RTW recommendations on a case-by-case basis	[88]
Söderman 2021	Oncology	Clinical units with joint routines/policies for handling sickness certification tasks (*n* = 342): 29.7%	[81]
Tsang 2020	Orthopedics	No use of guidelines, when advice was given, it was based on personal judgment only	[82]
van Velzen 2020	Rehabilitation	A Dutch guideline on acquired brain injury and RTW process is available but does not contain a protocol for a vocational rehabilitation intervention. Rehabilitation centers that use a local vocation rehabilitation protocol (*n* = 34): 59%	[89]
Walters 2010	General surgery, internal medicine, emergency medicine, general practice, orthopedics	Frequency of following a guideline when issuing sickness certificates by registered doctors in specialty training (*n* = 51): none, 71%; Department for Work and Pensions guidance, 2%; guideline from previous primary care trust, 15%; guideline from previous hospital department, 10%; and yes, unspecified, 2%	[84]

^a^Rowe 2018 and Gaudet 2019 reported on the same data; ^b^Lindholm 2010, Bränström 2013, and Ljungquist 2013 reported on the same data; ^c^Gustavsson 2013 and Gustavsson 2016 reported on the same data

*CI* confidence interval; *DMARDs* disease-modifying antirheumatic drugs; *IQR* interquartile range; *n* number; *OR* odds ratio; *RTW* return to work; *SD* standard deviation; *UK* United Kingdom; *wks* weeks

## Exploration of Work and Disease Work-Relatedness by Medical Specialists

### Collecting Information on a Patient’s Occupation

The initial step in exploring work or detecting work-related health problems is to collect information on a patient’s occupation, such as by asking about the patient’s current job or occupational status [38]. This practice varied from 10 to 93% [61, 82, 83, 87]. One study reported that orthopedic surgeons did not collect information on their patient’s occupation routinely nor in any standardized way [38]. In contrast, oncologists in another study were facilitated in collecting occupational data by an interview sheet [83].

### Establishing Work as the Cause of Injury or Disease

To make a proper diagnosis and treatment plan, it can be necessary to establish whether work is the cause of the injury or disease. According to medical specialists, when conducted within the context of injured workers’ compensation regulations, this can be an obligatory and difficult task [43, 53]. It can also be routine practice in many facilities [87]. To diagnose occupational disease, such as occupational asthma, history taking of workplace exposure and specific diagnostic tests are part of the clinical practice to establish work as the cause of disease [60, 70, 77].

### Exploring the Context of Work to Provide Advice

A qualitative study stated that the initial evaluation of a medical condition relies on a biomedical model and uses a disease-centered focus, whereas when work-related advice is needed details of the patient’s occupation must be explored and taken into account [53]. Although work was not the most important factor, several studies revealed that work-related factors also influenced medical decision-making [36, 38, 45, 65, 66, 105]. This was reflected in both the medical specialists’ advice as well as in the patients’ decisions to have certain treatment.

## Discussing Work-Related Concerns with Patients

### To Discuss or Not to Discuss Work-Related Concerns

Most studies that addressed this category reported that discussing work was not routinely embedded in clinical care [36, 37, 39, 44, 46, 48, 55, 56] ranging from 15 to 52% [47, 58, 95, 96]. One exception was found in rehabilitation care, in which 84% of the centers reported that obstacles for RTW were discussed in their center [87]. Furthermore, in recent years, some specialists have started to address work more frequently [44, 55] and 94–96% of the specialists provided RTW support when it was specifically asked for by a patient in to recent studies [72, 82].

### Timing of Discussing Work-Related Concerns

When work-related concerns were discussed, this could occur throughout all stages of care. Early recommendations have been shown to be critically important for those patients who receive them, since their clinical healthcare providers were the first point of contact to provide information on how to best proceed with work participation [50]. Yet, the timing of discussing work-related concerns ranged from early in treatment [44, 50, 53] to being first discussed during follow-up care [36, 41, 44, 46].

### Initiator of the Discussion About Work

All studies that addressed this category reported that conversations about work were started by the patient [35, 43, 44, 48] or reported that work-related advice was given when a patient asked for it [72, 82]. Patients who initiated the discussion included self-employed individuals [35], cancer survivors who asked for permission to RTW [44], patients who applied for insurance benefits and required medical information to provide to their insurer [48], and patients involved in worker injury claims [43].

## Nature of the Work-Related Advice Given to Patients

### Providing General Health Advice

Providing a patient with general health advice is a part of providing work-related advice, such as advice on the use of barrier creams in a case of occupational contact dermatitis [97] or advice on mental health for postpartum women in RTW consultations [98]. Although general health advice is part of work-related advice, it was sometimes shown to be the only advice being provided during work-related consultations. This was regarded by medical specialists as well as patients as insufficient and lacking the necessary practical details for work-related situations [35, 44, 48, 49]. Such advice entailed, for example, providing information on side effects, fatigue, or altered general performance [35, 44, 48] or advice to keep wounds dry, avoid heavy lifting and ‘do not force yourself’ [35, 79, 92].

### Patient–Physician Collaboration in Work-Related Advising

Some patients were guided by advice from the medical specialists [36, 78, 104]. However, decisions about work (e.g., when to RTW, whether to make work adjustments) were in the end dominated by the patients’ decisions or self-management strategies instead of being a result of collaboration with the physician [34–36, 45, 53, 69, 78, 106].

### Staying at Work and RTW Advice for Temporary Conditions and Post-operative Periods

Work-related advice for temporary conditions and post-operative periods focused on appropriate recovery time before RTW (in cases when ceasing work activities was temporary recommended or assumed) [35, 49, 64, 69, 78, 79, 85, 86, 88, 91, 99, 101, 102]. The judgment of whether or not a patient would be able to return to their current job following surgery was based on the physician’s perception of the physical job demands (i.e., desk based versus manual labor) [35, 38, 64, 79, 85, 86]. However, large discrepancies in RTW advice were shown between providers [78, 79, 85, 101].

Some studies mentioned that more individually tailored RTW advice depended on the situation of the patient, including recommendations regarding work adjustments (e.g., advice to temporarily cease specific activities that might aggravate a surgical wound or impact a reduced immune system) [35, 49, 64, 79, 88]. Others reported specialists paying attention to the patient’s perceptions and expectations toward their conditions and prognosis [38, 52, 53]. Patients who were limited in their work activities due to incapacity to drive to their workplace commonly received information on when they could return to driving [49, 64, 79]. For diseases with long treatment periods, the impact of treatment on work participation was explained by the specialist (e.g., time investment during oncological treatment) [42, 45, 54, 83]. However, from a patient’s point of view, this RTW advice was not always sufficiently tailored to their unique situation [49].

### Work-Related Advice for Chronic Diseases

Work-related advice for chronic diseases focused on preventing exacerbation by reducing or avoiding specific work exposures [46, 60, 70, 97] or coping with the disease in relation to work (e.g., recommending a reduction in work-related roles, to change work schedules, or to use assistive devices) [37, 50, 92]. Furthermore, for patients who started out with a temporary condition but who could not fully recover and became chronic patients, specialists might advise to consider changing or quitting their job if the job was physical demanding [38, 43, 100].

## Other Work-Related Support for Patients

### Scheduling Care Around Work

In some cases, work schedules were taken into account in treatment and appointment planning [38, 83]. However, other patients had to deal with hospital appointments conflicting with their working hours [37].

### Tailored Vocational Rehabilitation

Some rehabilitation clinics and hospitals offered dedicated vocational rehabilitation programs [87–89, 103]. However, not all patients were referred to rehabilitation programs which provided the vocational rehabilitation needed for RTW [36].

### Work-Related Support Guided by Systems for the Rehabilitation of Sick and Injured Workers

Two types of work-related support that were identified are embedded within national systems (i.e., systems for sickness certification and for rehabilitation of injured workers). Within these regulations, medical specialists—or physicians in general—provide sickness certificates or assess a patient’s work ability [41, 52, 54, 88]. However, some local hospital policies required that the patient must be referred to a general practitioner to get a sick note [90]. Sometimes sickness certification was handled as an administrative task and provided without a personal appointment with the patient [59, 80]. Specialists struggled with work ability assessments [41, 42] and sometimes assessed the work ability based more on the patient’s opinion than their professional opinion in order to maintain the patient’s trust [52]. Furthermore, it is not common for specialists to assess whether work adjustments can be made to enable RTW [90].

Most of the included studies on sickness certification were from Sweden [41, 42, 52, 59, 62, 63, 67, 68, 73–76, 80, 81, 93], where it used to be quite common for some specialists to write more than 20 sick notes per week [67, 68]. The next most common were studies from the UK [49, 58, 84, 90], followed by other countries such as Belgium, Canada, Japan, and the USA where it is also common for physicians to write sick notes for patients [48, 54, 69, 72, 83, 88]. In contrast, medical specialists play almost no role in sickness absence in the Netherlands [107]. Specific systems for the rehabilitation of injured workers were described in studies from Canada [43, 51] and the UK [53, 60].

## Interdisciplinary Cooperation Between Medical Specialists and Other Healthcare Providers

### Referral to Other Professionals

Several studies mentioned that physicians advised patients to seek support from other professionals for work-related problems [48, 53, 54, 59, 60, 62, 67, 77, 80, 97] or to involve the employer [60, 62, 79, 80, 83, 88]. These professionals included occupational health physicians [44, 54, 59, 60, 62, 67], professionals from specialized occupational disease clinics [77, 97], occupational therapists [48, 53, 62, 80], physical therapists [36, 53, 62, 80], psychologists [53, 62, 80], general practitioners [44], social workers [60, 62, 77, 80], and trade union representatives [60]. Although in some studies referral was recognized as useful, it was not always executed [36, 44, 48]. When patients were referred, information regarding RTW expectations was sometimes included in the correspondence [88] or patients were advised to tell their supervisor about treatment prospects and ask for support [83].

### Providing Work-Related Support in Collaboration with Other Professionals

Work-related support in collaboration with other professionals occurred with varying frequency in multidisciplinary teams or coordination meetings with different combinations of medical specialists, nurses, occupational therapists, physiotherapists, psychologists, social workers, social insurance agents, occupational health physicians, and employers [41, 42, 53, 62, 80, 83, 87, 89, 108]. To plan RTW, face-to-face meetings with all stakeholders were seen as important [42]. However, work-related support is not always on the agenda for multidisciplinary meetings that do not have a specific focus on work [44].

To reach stakeholders outside teams or without an established team, communication included written or telephone contact [42, 88, 89] or patients being used as ‘go-betweens’ among stakeholders [43, 87].

## Medical Guidelines Integrating Work and the Use of These Guidelines by Medical Specialists

Several studies mentioned that guidelines to provide work-related support were not available [35, 48, 53, 70, 77, 82, 99, 102, 106], which caused advice to be unreliable or not provided systematically to patients [35, 48, 53]. Instead, advice was based on personal judgment and expert opinion and included using other information resources, like journal articles [35, 53, 70, 82, 106]. In some institutions, local policies for providing work-related support, including sick listing, were available [69, 81, 88, 89]. For some patient groups or diseases, national guidelines were available [60, 64, 65, 68, 78, 79, 84, 86, 89, 97]. Medical specialists often were not aware of these guidelines (up to 100% of the sample in one study [64]) or did not follow or use them which ranged from 26 to 71% [68, 84]. Furthermore, some studies showed that there was not always consensus on the appropriate RTW advice within these guidelines [64, 78, 86].

## The extent to which policy documents and medical guidelines provides information on what medical specialists are obliged or recommended to do regarding the provision of CWIC

We describe the findings from the gray literature from a societal perspective (i.e., policy documents) and professional medical standards (i.e., medical guidelines) that provide information on what a medical specialist should do or is recommended to do regarding the provision of CWIC. The findings of the gray literature can be found in Table 4. The sixth category (i.e., medical guidelines integrating work and the use of these guidelines by medical specialists) was omitted, since medical guidelines themselves do not provide information on their use by medical specialists and the content of the guidelines is discussed in other main categories.

**Table 4:** Characteristics and findings of gray literature

Report name	Main finding(s)	n	Year	Source	References
**Vision documents**		2			
Seoul Statement on the Development of Occupational Health Services for All	–To ensure sufficient coordination and exchange within countries, continuous dialogue should be maintained and close and regular collaboration between occupational health and general health services	1	2015	ICOH	[109]
Toward age-friendly work in Europe: a life-course perspective on work and aging from EU Agencies	–Individual doctors need to have return to work as an outcome objective when treating patients	1	2017	Eurofound, EU-OSHA	[110]
**Policy reports**		26			
Occupational disease management					
Alert and sentinel approaches for the identification of work-related diseases in the EU (including Alert and sentinel systems: SIGNAAL, Netherlands/Belgium; SUVA, Switzerland; SENSOR-Pesticides Program, USA; THOR, United Kingdom)	–Medical specialists play a minor role in the reporting of occupational diseases –Physicians did not consider reporting to public health authorities a priority in their clinical practice	5	2018	EU-OSHA	[111–115]
Forum 11—Monitoring occupational safety and health in the European Union	–The UK OHS system uses voluntary reporting of occupational diseases by doctors –Medical doctors are given little awareness training in the problems of occupational diseases during their professional training. This should be addressed by mainstreaming OSH into their education	1	2004	EU-OSHA	[116]
Occupational skin diseases and dermal exposure in the European Union (EU-25): policy and practice overview	–In some European countries (e.g., Germany, Austria) dermatologist have a role in reporting occupational skin disease –Many workers with occupational skin disease see a dermatologist first. The dermatologist diagnoses the disease and provides treatment and advice on preventive measures –The role of the occupational health physician is to provide information to the dermatologist about the products thought to be responsible for the occupational skin disease	1	2008	EU-OSHA	[117]
Stay in work and return to work policies					
Rehabilitation and return to work: Analysis report on EU and Member States policies, strategies, and programs Safer and healthier work at any age Country Inventory^a^: Austria, Belgium, Bulgaria, Cyprus, Czech Republic, Denmark, Estonia, Finland, France, Germany, Greece, Ireland, Italy, Latvia, the Netherlands, Norway, Spain, and the UK	–Different policies exist between countries regarding rehabilitation and RTW, only some include a role for a medical specialist –In all countries, medical rehabilitation of the sick or injured worker takes place within the general healthcare system. In some countries with insurance-based systems, exceptions exist for workers suffering from occupational accidents or diseases who can follow medical treatment and rehabilitation in the facilities of insurance institutions –Medical doctors and occupational physicians have a pivotal role in the RTW process. In many countries, there is little or no coordination between medical doctors and the workplace, often as a result of medical confidentiality issues. In addition, in many countries, the lack of an appropriate cooperation structure significantly limits coordination between the primary physician and the medical experts of the organization coordinating the return to work process, such as a social security agency –In many European countries, a lack of coordination between medical doctors, vocational rehabilitation providers, and the workplace impedes or delays return to work –When they exist, coordination mechanisms intervene at different stages of the RTW process, starting at the very beginning of the process, when medical treatment is taking place, between medical doctors and the workplace (employer or occupational health services) –A characteristic of most RTW programs implemented by coordinating organization is their individualized approach. It starts with assessment of work capacity based on the principles of the biopsychosocial theoretical model, this assessment is likely to be done with the help of a multidisciplinary team, including medical, physical and mental health doctors, and therapists, but also social officers, vocational rehabilitation, and employment specialists	19	2015–2016	EU-OSHA	[118–136]
**Research reviews to inform later policy making**		3			
Biological agents and prevention of work-related diseases: a review	–In some European countries, a specialist network with dermatologists and pulmonologists exists to help detect occupational exposure of biological agents	1	2020	EU-OSHA	[137]
Musculoskeletal disorders in workers with multiple sclerosis: a task-oriented view	–To support workers with multiple sclerosis to prevent musculoskeletal disorders that needs a multidisciplinary approach, this multidisciplinary team usually includes a physician	1	2022	EU-OSHA	[138]
Research review on rehabilitation and return to work	–In the UK, a doctor considers the patient’s ability to work in general, based on revised guidance that reflects a move away from job-specific assessments and provides the patient with a ‘fit note’ –Coordination and cooperation between health and social security institutions and employers should be promoted	1	2016	EU-OSHA	[139]
**Medical guidelines**		24			
General					
Workplace health: long-term sickness absence and capability to work (UK)	–Guideline on how to help people return to work after long-term sickness absence, reduce recurring sickness absence, and help prevent people moving from short-term to long-term sickness absence	1	2020	GIN	[140]
Cardiology					
Acute coronary syndromes NICE guideline (UK)	Includes minor mentioning of work, that is, –Offer cardiac rehabilitation programs in a choice of venues and at a choice of times of day, for example, sessions outside of working hours –Take into account the physical and psychological status of the patient, the nature of their work and their work environment when giving advice on returning to work	1	2020	GIN	[141]
Neurology					
Clinical Practice Guideline for the Management of Patients with Parkinson’s Disease (Spain)	Includes minor mentioning of work, that is, –Includes work as a factor to consider in the management –When the patient’s work capacity is compromised, this justifies starting pharmacological treatment	1	2014	GIN	[142]
Obesity					
Management of Obesity in Adults (Qatar)	Includes minor mentioning of work, that is, –Sedentary and night-shift work listed as risk factors –Work-life balance listed as barrier to physical activity in management of obesity	1	2022	GIN	[143]
Oncology					
American Cancer Society/American Society of Clinical Oncology Breast Cancer Survivorship Care Guideline (USA)	Includes minor mentioning of work, that is, –Limited information on the impact of cancer to return to work support	1	2016	GIN	[144]
Blueprint cancer and occupation (in Dutch, the Netherlands)	–General guideline with regard to cancer and work for all phases of cancer targeted at different actors involved in treatment	1	2009	GIN	[145]
Breast cancer (in Dutch, the Netherlands)	Work is mentioned at several placed in this guideline: –Work/occupations should be mentioned during treatment –Refers to guideline ‘Blueprint cancer and occupation’	1	2012	GIN	[146]
	Work is mentioned at several places in this guideline: –Work/occupation should be mentioned during treatment –Mentions that one common complication has impact on work –Section on support for RTW –Refers to an update of guideline ‘Blueprint cancer and occupation’	1	2020	GIN	[147]
Hepatocellular carcinoma (in Dutch, the Netherlands)	Includes minor mentioning of work, that is, –Refers to guideline ‘Blueprint cancer and occupation’	1	2013	GIN	[148]
Intracranial meningioma (in Dutch, the Netherlands)	Includes minor mentioning of work, that is, –Mentions that work is covered as part of rehabilitation	1	2015	GIN	[149]
Screening for psychosocial distress (in Dutch, the Netherlands)	–General guideline on screening for psychosocial distress in cancer patients (including work)	1	2017	GIN	[150]
Ophthalmology					
Clinical Practice Guideline for the Management of Glaucoma (Malaysia)	Includes a key recommendation on work, that is, - Patients should be referred to rehabilitation including vocational rehabilitation	1	2017	GIN	[151]
Respiratory disease					
Clinical Practice Guideline: Allergic Rhinitis (USA)	Includes minor mentioning of work, that is, –Impairment of work listed as factor of disease classification as more severe	1	2015	GIN	[152]
Cough: Occupational and Environmental Considerations – ACCP Evidence-Based Clinical Practice Guidelines (Canada)	–Guideline on the role of occupational and environmental factors in causing and contributing to cough	1	2006	GIN	[153]
Managing the long-term effects of COVID-19 (UK)	Work is mentioned throughout this guideline included as follows: –Advice to discuss the effect of symptoms on work –RTW advice –Use an ability to return to usual activities, including work, as measure of recovery –Provide integrated, multidisciplinary rehabilitation services, including support to work –Refers also to guideline ‘Workplace health: long-term sickness absence and capability to work’	1	2022	GIN	[154]
The Assessment & Management of Chronic Obstructive Pulmonary disease in Adults (Qatar)	Includes minor mentioning of work, that is, –During history taking to assess the impact on patient’s life, including missed work and socioeconomic impact	1	2020	GIN	[155]
The Diagnosis and Management of Asthma in Adults (Qatar)	Includes minor mentioning of work, that is, –During history taking to elicit information about: Materials with which they work. Whether their symptoms improve regularly when away from work –Provide information that 1 in 6 cases of new or recurrent asthma is attributable to occupation –To consider Occupational Asthma –During monitoring to include asking about time off work	1	2019	GIN	[156]
Rehabilitation					
Brain injury rehabilitation in adults (UK)	–Includes a chapter about vocational rehabilitation	1	2013	GIN	[157]
Cancer rehabilitation (in Dutch, the Netherlands)	–Includes a chapter about work participation after cancer	1	2013	GIN	[158]
Cardiac rehabilitation (UK)	–Includes a chapter about vocational rehabilitation	1	2017	GIN	[159]
Clinical Practice Guideline for the Rehabilitation of Adults with Moderate to Severe TBI (Traumatic Brain Injury) (Canada)	–Includes a section about vocational rehabilitation	1	2016	GIN	[160]
Rehabilitation after critical illness in adults Clinical guideline (UK)	Includes minor mentioning of work, that is, –To give patients information before their discharge to home or community care (if applicable) about driving, returning to work, housing, and benefits	1	2009	GIN	[161]
Rehabilitation for persons with traumatic brain injury (USA)	–Includes a chapter about rehabilitation for work to help a person return to work after a brain injury	1	2004	WHO	[162]
Stroke rehabilitation in adults Clinical guideline (UK)	–Includes a section about RTW –Refers also to guideline ‘Workplace health: long-term sickness absence and capability to work’	1	2013	GIN	[163]

*EU-OSH* European Agency for Safety and Health at Work; *GIN* Guidelines International Network; *ICOH* International Commission on Occupational Health; *n* number; *OHS* Occupational Health and Safety; *OR* odds ratio; *UK* United Kingdom; *USA* United States of America; *WHO* World Health Organization

^a^In total, 30 country inventories were made by the EU-OSHA. However, the excluded documents had no reference to a medical specialist within these documents

## Exploration of Work and Disease Work-Relatedness by Medical Specialists

In some countries (e.g., UK, Germany, Austria), medical specialists are expected to a play a voluntary role in exploring and reporting occupational diseases [111, 116, 117, 137]. As a consequence of being voluntary, medical specialists do not always prioritize reporting occupational diseases to public health authorities [113]. Several guidelines state that a medical specialist should explore work when taking down a patient’s history to detect work-exposure risks early [143, 153, 156]. Furthermore, guidelines recommended that work impairments be explored to classify disease severity [152] or to start treatment [142].

## Discussing Work-Related Concerns with Patients

One vision document stated that individual doctors need to have RTW as an outcome objective when treating patients [110], implying that work should be discussed. Furthermore, several guidelines stated that a medical specialist should address work throughout all treatment phases to assess vocational rehabilitation needs early on to stimulate work participation and prevent delay in RTW [140, 142, 145–148, 150, 151, 154, 155].

## Nature of the Work-Related Advice Given to Patients

Although medical specialists are expected to provide work-related advice to patients, information on the nature of this advice is limited in guidelines and does not include details in terms of days to RTW [144, 154, 161]. Furthermore, instead of including specific information themselves, several national guidelines [146–148, 154, 163] refer to two comprehensive guide dedicated to supporting people to RTW [140] and supporting work participation in cancer treatment [145], respectively.

## Other Work-Related Support for Patients

To encourage people to attend cardiac rehabilitation, one guideline recommended scheduling care around the working lives of patients [141]. Rehabilitation guidelines themselves often state that patients should be provided with information about work participation after rehabilitation [158, 161] or include a dedicated chapter on vocational rehabilitation [157, 159, 160, 162, 163].

A subcategory which was not identified from the bibliographic database search was prevention of work-related disease. This topic was covered by one research review, which stated that it is necessary to organize a multidisciplinary team, including a physician to support workers with multiple sclerosis to prevent developing musculoskeletal disorders [138].

## Interdisciplinary Cooperation Between Medical Specialists and Other Healthcare Providers

In the RTW rehabilitation process, medical specialists are one group among many other stakeholders (as outlined by many European policy reports, a research review, and one guideline) [118–136, 139, 145]. Coordination between these stakeholders should be maintained and encouraged, since a lack of coordination between medical doctors, vocational rehabilitation providers, and the workplace impedes or delays RTW [109, 118]. However, in many countries, there is little or no structure embedded within the systems to serve such cooperation, which limits the cooperation between the stakeholders [118]. Several medical guidelines did recommend referring patients to vocational rehabilitation or occupational health care [147–149, 151].

## Discussion

This study aimed to systematically map the extent and nature to which medical specialists provide clinical work-integrating care (CWIC) and to reflect on what policy documents and medical guidelines oblige or recommend the medical specialist to do regarding the provision of CWIC. Asking a patient about work and discussing work-related concerns were embedded in clinical practice to varying extents and, in many countries, embedded within legislation. Work-related advice was often based on personal judgment and expert opinion because guidelines lacked evidence were not available or were not used. The work-related advice that was provided covered general health advice or RTW advice without including practical details for work-related situations. Decisions about work (e.g., when to RTW, whether to make work adjustments) were usually primarily based on the patient’s judgments or self-management strategies instead of being a result of collaboration with the physician. Yet, sometimes patients were referred to other professionals or vocational rehabilitation for work-related support.

Policy documents reported that medical specialists should provide some degree of CWIC [109, 110, 116]. Consistent with this demand, a few recent studies within our review reported that work-related conversations between medical specialists and patients occurred regularly [44, 55, 72, 82]. Yet, this still was often found not to be the current practice [36–39, 44, 46–48, 55, 56, 58, 82, 83, 95, 96]. Furthermore, all studies included in this review that reported on the question of who initiated the conversation about work stated that these conversations were dependent on the initiative of the patient [35, 43, 44, 48, 72, 82]. From the perspective of hospital-based health care, this could be explained by the fact that historically the domain of occupational health and safety was not allocated to clinical health care [164]. In many countries, occupational health care was not part of primary care either, and general practitioners similarly reported that they sometimes inquire about the work of the patient but do not document this [165]. This historical position of work-related care as the sole domain of occupational health and safety could also be the reason why medical specialists are not always educated during medical training on work-related topics, as many specialists report [43, 48, 57, 61, 69, 84, 116, 166]. This lack of expertise and knowledge of occupational health and safety has been similarly indicated by general practitioners [165, 167–171]. Furthermore, it could explain the limited information from guidelines that we found in this review [35, 48, 53, 70, 77, 82, 99, 102, 106, 144]. Both lack of education and lack of guidelines have influence on the specialists’ work-related advice, which according to medical specialists as well as patients is often insufficient due to lack of practical applications fit for work-related situations [35, 44, 48, 49]. However, patients require personally tailored information to support them in their work-related goals as an outcome measure of good health [36, 37, 46, 49, 52, 172, 173].

During the gray literature search, we included most policy documents and medical guidelines from the last seven years, because older documents and guidelines did not mention aspects of CWIC that should be provided by medical specialists. This could be explained by an altered paradigm of health care in which health is increasingly regarded as the ability to adapt to the physical, emotional, and social challenges of life instead of merely the absence of pathology [174, 175]. Within this paradigm, health is not defined by a physician but by the person according to his or her functional needs. Secondly, more recent changes in the demographics of workforces makes it increasingly important for policy makers to take measures to maintain health, since the relative number of working patients will globally increase in the coming decades [1, 8, 176, 177]. Therefore, policy makers ask for more attention toward work from clinical health care [10, 18, 20, 22, 109, 110].

Furthermore, our results show that work-related guidance is frequently embedded within legislation, such as regulations on sick leave or worker injury [41, 52, 54, 88, 118]. Such national regulations could serve to stimulate more conversations about work [20, 52], because they could provide financial means to increase the number of healthcare providers that support patients with their work participation. However, regulations also come with challenges of integrating the topic of work into clinical health care. Firstly, regulations can trigger tension in the physician–patient relationship [48, 52]. Insurance agencies often play a role in executing regulations and can have different interests and perspectives than patients have (i.e., differing opinions about the patient’s capacity to RTW which influences whether the patient is eligible for disability benefits). This places the medical specialist in a difficult position when he or she is obliged to make a recommendation about work ability. Furthermore, the nature of the information that insurers can request is not always regarded to be within the medical specialists’ mandate of care [43, 48, 51, 53]. For example, the insurer may ask how many pounds a patient can lift of an oncologist, which is not within the scope of interest for oncological treatment [48]. This can result in a specialist responding negatively toward the insurer regarding the patient’s work capacity and thereby negatively affect the patient’s work integration [43, 48, 51, 53]. Moreover, to support patients with work within regulations, medical specialists need other professionals to refer their patients because providing the full scope of work-related support is beyond most specialists’ expertise [53, 54]. However, communication between all professionals who play a role within these regulations is often difficult, since it is hard to reach the others by telephone [43, 51, 87]. In summary, acting in accordance with legislation has become a difficult task for many specialists, which hinders making work participation an important treatment goal [41–43, 51, 52, 90].

### Methodological Considerations

We performed a comprehensive search for this scoping review using different perspectives to examine the extent and nature of providing CWIC and what is obliged or recommended regarding CWIC in current practice. By doing so, we could review the full scope of how medical specialists provide CWIC and augmented this with what specialists are obliged or recommended to do as described in policies and medical guidelines. This resulted in a heterogeneous assembly of findings. Using a qualitative analysis technique, we were able to structure the different perspectives and outcomes into a coherent narrative.

Our final search resulted in more than 20,000 results and screening and selection of these many hits was prone to selection bias. To reduce this bias, we performed all screening phases in duplicate. We attempted to reduce the number of hits by trying out different combinations of words in our search strategy and applying filters, since we were not interested in aggregated evidence, such as systematic reviews. However, to detect all studies we were beforehand familiar with a sensitive search was needed. This resulted in the comprehensive search we finally used for this scoping review.

During the gray literature search, we selected our sources based on our own expert opinion and mainly focused on European documents. However, we were limited by language restrictions and therefore, it is likely that we missed several important documents in languages other than Dutch and English. We especially suspect this bias with the guidelines. This could have impacted our findings, because in other countries, the professional standard might be different (for example, because legislation provides no incentive to include RTW as outcome objective in clinical care) [129]. Although we suspect bias in the gray literature by limiting our sources and languages, adding the gray literature allowed for triangulation. This let us combine data sources and review the concept of CWIC from different perspectives by exploring our two research questions. From there, we could understand that in different countries, different regulations exist, which in turn have differing impact on the extent to which medical specialists provide CWIC or are obliged to do so.

The information the different evidence provided ranged from a single useable quote or outcome to a complete study about our topic of interest. Although a single quote will only have informed one subcategory, this imbalance must be considered when interpreting our results. Therefore, we provided a list of studies which covered each category (Table 2) to guide the reader with this interpretation. Reviewing this table, we suspect an underreporting in the literature on the extent to which medical specialists practice CWIC. For example, to discuss work-related concerns with a patient, a specialist will probably start by asking what occupation the patient has. Yet, studies reporting on having discussions about work did not all report on collecting occupational information (most likely due to their different study aims). This will not have affected our findings on the nature of CWIC, but more evidence on the extent to which CWIC is provided needs to be explored and reported in literature.

### Implications for Practice and Further Research

Our results show that actual discussions about work with patients are lagging behind the growing demand from policies and guidelines to provide some degree of CWIC in clinical health care. Furthermore, conversations about work are often initiated by the patient. However, there is an increasing understanding among medical specialists about the importance of work in recent years. Attention to work participation from the start of a diagnosis is important, because it results in better outcomes for patients, employers, and hospital physicians [3, 42, 178, 179]. Additionally, when patients receive work-related guidance, they are better empowered to make decisions related to work participation problems [50, 180].

In many countries, healthcare systems are not designed for optimal attention to work in clinical health care, which is a barrier for CWIC. National regulations to reduce sick leave actually hinder a medical specialist’s ability to be open in a conversation about work due to fear of tension in the patient–physician relationship [10, 48, 52]. However, these national regulations can also work as a stimulant for conversations about work [20, 52], because they could provide financial means to increase the numbers of healthcare providers supporting patients with their work participation. It is key to improve the coordination and cooperation between all involved professionals [43, 51, 109, 118–136, 139]. How the existing regulations can best be used and what their corresponding limitations are will differ between countries [181]. Unraveling this question could be a topic of further research, within which the influence of jurisdiction in different countries on provision and content of CWIC might also be explored.

For an individual specialist to provide CWIC, knowledge and medical training are needed. Knowledge could be captured within clinical guidelines [68, 81]. However, it is known that it might take decades for healthcare professionals to apply new evidence [182]. Future research should focus on the aggregation of evidence in guidelines and guideline implementation strategies [68, 93, 178]. This research might also reveal critical knowledge gaps. Furthermore, medical training for medical specialists is needed to support patients in healthy work participation. This training should focus on the importance of work as a clinical outcome [61, 166, 183], occupational disease [70, 77, 116], applying local regulations [54, 57, 62, 68, 84], and supporting patients in their personal coping with challenges regarding specific work-related situations [34, 40, 50, 108].

## Conclusion

Medical specialists often provide some degree of CWIC. This may range from asking about the patient’s occupation to extensive collaboration with the patient or other healthcare professionals to support work participation. Yet, conversations about work participation are not routinely embedded in clinical practice and are mostly initiated by the patient. Furthermore, medical guidelines are often limited in their availability or lacking useful information about what to advise patients about work. In recent years, society has increasingly expected medical specialists to provide some attention to work participation (e.g., by collaborating with occupational healthcare specialists or include RTW as treatment outcome), which is in line with recent studies that show an increasing tendency to discuss work between patients and medical specialists. Due to the expected rise in the number of people of working age with a disease, integrating work into clinical health care becomes increasingly important to sustain a healthy workforce in the future.

### Supplementary Information

Below is the link to the electronic supplementary material.

Supplementary file1 (DOCX 23 KB)

Supplementary file2 (DOCX 13 KB)

Supplementary file3 (DOCX 69 KB)
