# Supplementary material for: Clinical Work-Integrating Care in Current Practice: A Scoping Review
Source: J Occup Rehabil. 2023 Nov 15;34(3):481–521. doi: 10.1007/s10926-023-10143-1 (PMC11364593; doi:10.1007/s10926-023-10143-1)
Supplement: Supplementary file 1 — Supplementary file1 (DOCX 23 KB) [file 10926_2023_10143_MOESM1_ESM.docx]

## Supplemantary file 1 Search strategy

### Pubmed

(("health care professional"[tiab] OR "health care professionals"[tiab] OR "healthcare professional"[tiab] OR "healthcare professionals"[tiab] OR "healthcare provider"[tiab] OR "healthcare providers"[tiab] OR "health care providers"[tiab] OR "health care providers"[tiab] OR "physician"[tiab] OR "physicians"[tiab] OR "Physicians"[Mesh] OR "Physicians, Women"[Mesh] OR "medical specialist"[tiab] OR "medical specialists"[tiab])) OR ("allergist"[tiab] OR "allergists"[tiab] OR "Allergists"[Mesh] OR "anesthesiologist"[tiab] OR "anesthesiologists"[tiab] OR "Anesthesiologists"[Mesh] OR "cardiologist"[tiab] OR "cardiologists"[tiab] OR "Cardiologists"[Mesh] OR "dermatologist"[tiab] OR "dermatologists"[tiab] OR "Dermatologists"[Mesh] OR "endocrinologist"[tiab] OR "endocrinologists"[tiab] OR "Endocrinologists"[Mesh] OR "gastroenterologist"[tiab] OR "gastroenterologists"[tiab] OR "Gastroenterologists"[Mesh] OR "hepatologist"[tiab] OR "hepatologists"[tiab] OR "gyneacologist"[tiab] OR "gyneacologists"[tiab] OR "gynecologist"[tiab] OR "gynecologists"[tiab] OR "hematologist"[tiab] OR "hematologists"[tiab] OR "haematologist"[tiab] OR "haematologists"[tiab] OR "hospitalist"[tiab] OR "hospitalists"[tiab] OR "Hospitalists"[Mesh] OR "immunologist"[tiab] OR "immunologists"[tiab] OR "infectiologist"[tiab] OR "infectiologists"[tiab] OR "internist"[tiab] OR "internists"[tiab] OR "nephrologist"[tiab] OR "nephrologists"[tiab] OR "Nephrologists"[Mesh] OR "neurologist"[tiab] OR "neurologists"[tiab] OR "Neurologists"[Mesh] OR "neurosurgeon"[tiab] OR "neurosurgeons"[tiab] OR "Neurosurgeons"[Mesh] OR "oncologist"[tiab] OR "oncologists"[tiab] OR "Oncologists"[Mesh] OR "ophthalmologist"[tiab] OR "ophthalmologists"[tiab] OR "Ophthalmologists"[Mesh] OR "Orthopedic Surgeons"[Mesh] OR "otolaryngologist"[tiab] OR "otolaryngologists"[tiab] OR "Otolaryngologists"[Mesh] OR "physiatrist"[tiab] OR "physiatrists"[tiab] OR "Physiatrists"[Mesh] OR "pulmonologist"[tiab] OR "pulmonologists"[tiab] OR "Pulmonologists"[Mesh] OR "Radiation Oncologists"[Mesh] OR "radiologist"[tiab] OR "radiologists"[tiab] OR "Radiologists"[Mesh] OR "radiotherapist"[tiab] OR "radiotherapists"[tiab] OR "rheumatologist"[tiab] OR "rheumatologists"[tiab] OR "Rheumatologists"[Mesh] OR "surgeon"[tiab] OR "surgeons"[tiab] OR "Surgeons"[Mesh] OR "traumatologist"[tiab] OR "traumatologists"[tiab] OR "urologist"[tiab] OR "urologists"[tiab] OR "Urologists"[Mesh])

AND

("absenteeism"[Mesh] OR "absenteeism"[tiab] OR "disability management"[tiab] OR "employability"[tiab] OR "employable"[tiab] OR "employment"[Mesh] OR "employment"[tiab] OR "Occupational Health Services"[Mesh] OR "occupational health"[Mesh] OR "occupational medicine"[Mesh] OR "occupational therapy"[Mesh] OR "Occupations"[Mesh] OR "occupation"[tiab] OR "occupations"[tiab] OR "rehabilitation, vocational"[Mesh] OR "return to work"[Mesh] OR "return to work"[tiab] OR "sick leave"[Mesh] OR "sick leave"[tiab] OR "sickness absence"[tiab] OR "unemployed"[tiab] OR "unemployment"[Mesh] OR "unemployment"[tiab] OR "vocational guidance"[Mesh] OR "vocational"[tiab] OR "work ability"[tiab] OR "work activity"[tiab] OR "work capacity"[tiab] OR "work disability"[tiab] OR "work participation"[tiab] OR "work rehabilitation"[tiab] OR "work retention"[tiab] OR "work status"[tiab] OR "work"[Mesh] OR "workability"[tiab] OR ("labour"[tiab] OR ("labor"[tiab] OR "work"[tiab]) AND market[tiab] AND entry[tiab]))

AND

("advice"[tiab] OR "advising"[tiab] OR "assisting"[tiab] OR "consultation"[tiab] OR "consultations"[tiab] OR "Counseling"[Mesh] OR "Decision Making"[Mesh] OR "discussing implications"[tiab] OR "discussing options"[tiab] OR "guidance"[tiab] OR "inform"[tiab] OR "information"[tiab] OR "Patient-Centered Care"[Mesh] OR "recommendation"[tiab] OR "recommendations"[tiab] OR "refer"[tiab] OR "referral"[tiab] OR "support"[tiab])

NOT (animals [mh] NOT humans [mh])

Filters: Publication date from 2005/01/01; English; Dutch

### Embase

| 1 | (healthcare professional* or health care professional*).mp |
| --- | --- |
| 2 | (healthcare provider* or health care provider*).mp |
| 3 | *physician/ or physician*.mp |
| 4 | exp medical specialist/ or medical specialist*.mp |
| 5 | exp immunologist/ or (immunologist* or allergist*).mp |
| 6 | exp anesthesiologist/ or anesthesiologist*.mp |
| 7 | exp cardiologist/ or cardiologist*.mp |
| 8 | exp dermatologist/ or dermatologist*.mp |
| 9 | exp endocrinologist/ or endocrinologist*.mp |
| 10 | exp gastroenterologist/ or gastroenterologist*.mp |
| 11 | exp gynecologist/ or (gynecologist* or gynaecologist*).mp |
| 12 | exp hematologist/ or exp hematologist-oncologist/ or (hematologist* or haematologist*).mp |
| 13 | exp medical staff/ or hospitalist*.mp |
| 14 | exp infectious disease specialist/ or infectiologist*.mp |
| 15 | exp nephrologist/ or nephrologist*.mp |
| 16 | exp neurologist/ or neurologist*.mp |
| 17 | exp neurosurgeon/ or neurosurgeon*.mp |
| 18 | exp medical oncologist/ or exp radiation oncologist/ or exp oncologist/ or oncologist*.mp |
| 19 | exp ophthalmologist/ or ophthalmologist*.mp |
| 20 | exp orthopedic surgeon/ or orthopedic surgeon*.mp |
| 21 | exp otolaryngologist/ or otolaryngologist*.mp |
| 22 | exp physiatrist/ or physiatrist*.mp |
| 23 | exp pulmonologist/ or pulmonologist*.mp |
| 24 | exp radiologist/ or exp interventional radiologist/ or radiologist*.mp |
| 25 | exp radiotherapist/ or radiotherapist*.mp |
| 26 | exp rheumatologist/ or rheumatologist*.mp |
| 27 | exp surgeon/ or surgeon*.mp |
| 28 | exp urologist/ or urologist*.mp |
| 29 | traumatologist*.mp |
| 30 | exp hepatologist/ or hepatologist*.mp |
| 31 | exp internist/ or internist*.mp |
| 32 | 1 or 2 or 3 or 4 or 5 or 6 or 7 or 8 or 9 or 10 or 11 or 12 or 13 or 14 or 15 or 16 or 17 or 18 or 19 or 20 or 21 or 22 or 23 or 24 or 25 or 26 or 27 or 28 or 29 or 30 or 31 |
| 33 | exp return to work/ or exp work resumption/ or return to work.mp |
| 34 | exp employment/ or exp employment status/ or employment.mp |
| 35 | exp unemployment/ or (unemployment or unemployed).mp |
| 36 | (absenteeism or sick leave or sickness absence).mp |
| 37 | labor market entry.mp |
| 38 | vocational*.mp |
| 39 | exp vocational rehabilitation/ or work rehabilitation.mp |
| 40 | (work ability or workability).mp |
| 41 | exp work capacity/ or work capacity.mp |
| 42 | exp work disability/ or work disability.mp |
| 43 | work status.mp |
| 44 | work retention.mp |
| 45 | employability.mp |
| 46 | employable.mp |
| 47 | employee*.mp |
| 48 | occupation.mp |
| 49 | disability management.mp |
| 50 | 33 or 34 or 35 or 36 or 37 or 38 or 39 or 40 or 41 or 42 or 43 or 44 or 45 or 46 or 47 or 48 or 49 |
| 51 | exp patient guidance/ or exp counseling/ or (guidance or counseling).mp |
| 52 | (advice or advising).mp |
| 53 | assisting.mp |
| 54 | consultation*.mp |
| 55 | decision making.mp |
| 56 | discussing implications.mp |
| 57 | discussing options.mp |
| 58 | exp patient information/ or inform.mp |
| 59 | patient-centered care.mp |
| 60 | recommendation*.mp |
| 61 | exp patient referral/ or (referral or refer).mp |
| 62 | support.mp |
| 63 | 51 or 52 or 53 or 54 or 55 or 56 or 57 or 58 or 59 or 60 or 61 or 62 |
| 64 | (exp animal/ or nonhuman/) not exp human/ |
| 65 | 32 and 50 and 63 |
| 66 | 65 not 64 |
| 67 | limit 66 to ((dutch or english) and yr="2005 -Current") |

### Psychinfo

| 1 | (healthcare professional* or health care professional*).mp |
| --- | --- |
| 2 | (healthcare provider* or health care provider*).mp |
| 3 | *Physicians/ or physician*.mp |
| 4 | medical specialist*.mp |
| 5 | (immunologist* or allergist*).mp |
| 6 | anesthesiologist*.mp |
| 7 | cardiologist*.mp |
| 8 | dermatologist*.mp |
| 9 | endocrinologist*.mp |
| 10 | gastroenterologist*.mp |
| 11 | exp gynecologist/ or (gynecologist* or gynaecologist*).mp |
| 12 | (hematologist* or haematologist*).mp |
| 13 | hospitalist*.mp |
| 14 | infectiologist*.mp |
| 15 | nephrologist*.mp |
| 16 | exp neurologist/ or neurologist*.mp |
| 17 | exp neurosurgeon/ or neurosurgeon*.mp |
| 18 | oncologist*.mp |
| 19 | ophthalmologist*.mp |
| 20 | orthopedic surgeon*.mp |
| 21 | otolaryngologist*.mp |
| 22 | physiatrist*.mp |
| 23 | pulmonologist*.mp |
| 24 | radiologist*.mp |
| 25 | radiotherapist*.mp |
| 26 | rheumatologist*.mp |
| 27 | exp surgeon/ or surgeon*.mp |
| 28 | urologist*.mp |
| 29 | traumatologist*.mp |
| 30 | hepatologist*.mp |
| 31 | exp internist/ or internist*.mp |
| 32 | 1 or 2 or 3 or 4 or 5 or 6 or 7 or 8 or 9 or 10 or 11 or 12 or 13 or 14 or 15 or 16 or 17 or 18 or 19 or 20 or 21 or 22 or 23 or 24 or 25 or 26 or 27 or 28 or 29 or 30 or 31 |
| 33 | exp reemployment/ or return to work.mp |
| 34 | exp employment/ or exp employment status/ or employment.mp |
| 35 | exp unemployment/ or (unemployment or unemployed).mp |
| 36 | (absenteeism or sick leave or sickness absence).mp |
| 37 | labor market entry.mp |
| 38 | vocational*.mp |
| 39 | exp vocational rehabilitation/ or work rehabilitation.mp |
| 40 | (work ability or workability).mp |
| 41 | work capacity.mp |
| 42 | work disability.mp |
| 43 | work status.mp |
| 44 | work retention.mp |
| 45 | employability.mp |
| 46 | employable.mp |
| 47 | employee*.mp |
| 48 | exp occupations/ or occupation.mp |
| 49 | disability management.mp |
| 50 | 33 or 34 or 35 or 36 or 37 or 38 or 39 or 40 or 41 or 42 or 43 or 44 or 45 or 46 or 47 or 48 or 49 |
| 51 | exp occupational guidance/ or exp counseling/ or (guidance or counseling).mp |
| 52 | (advice or advising).mp |
| 53 | assisting.mp |
| 54 | consultation*.mp |
| 55 | decision making.mp. or exp Decision Making/ |
| 56 | discussing implications.mp |
| 57 | discussing options.mp |
| 58 | inform.mp |
| 59 | patient-centered care.mp |
| 60 | recommendation*.mp |
| 61 | (referral or refer).mp |
| 62 | support.mp |
| 63 | 51 or 52 or 53 or 54 or 55 or 56 or 57 or 58 or 59 or 60 or 61 or 62 |
| 64 | (animal not human).po |
| 65 | 32 and 50 and 63 |
| 66 | 65 not 64 |
| 67 | limit 66 to ((dutch or english) and yr="2005 -Current") |

### CINAHL

| S1 | "healthcare professional*" or "health care professional*" |
| --- | --- |
| S2 | "healthcare provider*" or "health care provider*" |
| S3 | (MM "Physicians") or (MM "Physicians, Women") or "physician*" |
| S4 | "medical specialist*" |
| S5 | (MM "Allergists") or "allergist*" or "immunologist*" |
| S6 | (MM "Anesthesiologists") or "anesthesiologist*" |
| S7 | (MM "Cardiologists") or "cardiologist*" |
| S8 | (MM "Dermatologists") or "dermatologist*" |
| S9 | (MM "Endocrinologists") or "endocrinologist*" |
| S10 | (MM "Gastroenterologists") or "gastroenterologist*" or "hepatologist*" |
| S11 | "gyneacologist*" or "gynecologist*" |
| S12 | "hematologist*" or "haematologist*" |
| S13 | (MM "Hospitalists") or "hospitalist*" |
| S14 | "infectiologist*" |
| S15 | "internist*" |
| S16 | (MM "Nephrologists") or "nephrologist*" |
| S17 | (MM "Neurologists") or "neurologist*" |
| S18 | "neurosurgeon*" |
| S19 | (MM "Oncologists") or "oncologist*" |
| S20 | (MM "Ophthalmologists") or "ophthalmologist*" |
| S21 | (MM "Otolaryngologists") or "otolaryngologist*" |
| S22 | "Orthopedic Surgeon*" |
| S23 | (MM "Physiatrists") or "physiatrist*" |
| S24 | (MM "Physicians, Emergency") |
| S25 | (MM "Pulmonologists") or "pulmologist*" |
| S26 | (MM "Radiation Oncologists") or "Radiation Oncologists*" or "radiotherapist*" |
| S27 | (MM "Radiologists") or "radiologist*" |
| S28 | (MM "Rheumatologists") or "rheumatologist*" |
| S29 | (MM "Surgeons") or "surgeon*" |
| S30 | "traumatologist*" |
| S31 | (MM "Urologists") or "urologist*" |
| S32 | S1 OR S2 OR S3 OR S4 OR S5 OR S6 OR S7 OR S8 OR S9 OR S10 OR S11 OR S12 OR S13 OR S14 OR S15 OR S16 OR S17 OR S18 OR S19 OR S20 OR S21 OR S22 OR S23 OR S24 OR S25 OR S26 OR S27 OR S28 OR S29 OR S30 OR S31 |
| S33 | (MH "Job Re-Entry") OR "return to work" |
| S34 | (MH "Employment") OR (MH "Employment of Disabled") OR (MH "Employment of Older Workers") OR (MH "Employment, Supported") |
| S35 | "employment" or '"employability" or "employable" or "employee*" |
| S36 | (MH "Unemployment") OR "unemployment" or "unemployed" |
| S37 | (MH "Absenteeism") OR "absenteeism" OR (MH "Sick Leave") OR "sick leave" OR "sickness absence" |
| S38 | (MH "Rehabilitation, Vocational") OR "work rehabilitation" |
| S39 | "vocational*" |
| S40 | "work ability" or "workability" or "work capacity" |
| S41 | "work disability" |
| S42 | "work status" |
| S43 | "work retention" |
| S44 | "work activity" |
| S45 | "work participation" |
| S46 | "labor market entry" or "labour market entry" |
| S47 | (MH "Occupations and Professions") |
| S48 | "occupation*" |
| S49 | S33 OR S34 OR S35 OR S36 OR S37 OR S38 OR S39 OR S40 OR S41 OR S42 OR S43 OR S44 OR S45 OR S46 OR S47 OR S48 |
| S50 | (MH "Vocational Guidance") OR "vocational guidance" OR "occupational guidance" |
| S51 | (MH "Counseling") or "counseling" or "guidance" |
| S52 | "advice" or "advising" |
| S53 | "assisting" |
| S54 | (MH "Referral and Consultation+") OR "consultation*" OR "refer" OR "referral" |
| S55 | (MH "Decision Making+") OR "decision making" OR (MH "Decision Making, Clinical") OR (MH "Decision Making, Shared") OR (MH "Decision Making, Patient+") |
| S56 | "discussing implications" |
| S57 | "discussing options" |
| S58 | "inform" or "information" |
| S59 | (MH "Patient Centered Care") OR "patient centered care" |
| S60 | "recommendation*" |
| S61 | "support" |
| S62 | S50 OR S51 OR S52 OR S53 OR S54 OR S55 OR S56 OR S57 OR S58 OR S59 OR S60 OR S61 |
| S63 | (MH "Animals" NOT MH "Human) |
| S64 | S32 AND S49 AND S62 |
| S65 | S64 NOT S63 |
| S66 | Filter date and language |

### Web of Science

#1 TS=("health care professional") OR TS=("health care professionals") OR TS=("healthcare professional") OR TS=("healthcare professionals") OR TS=("healthcare provider") OR TS=("healthcare providers") OR TS=("health care provider") OR TS=("health care providers") OR TS=("physician") OR TS=("physicians") OR TS=("medical specialist") OR TS=("medical specialists") OR TS=("allergist") OR TS=("allergists") OR TS=("anesthesiologist") OR TS=("anesthesiologists") OR TS=("cardiologist") OR TS=("cardiologists") OR TS=("dermatologist") OR TS=("dermatologists") OR TS=("endocrinologist") OR TS=("endocrinologists") OR TS=("gastroenterologist") OR TS=("gastroenterologists") OR TS=("hepatologist") OR TS=("hepatologists") OR TS=("gyne$cologist") OR TS=("gyne$cologists") OR TS=("he$matologist") OR TS=("he$matologists") OR TS=("hospitalist") OR TS=("hospitalists") OR TS=("immunologist") OR TS=("immunologists") OR TS=("infectiologist") OR TS=("infectiologists") OR TS=("internist") OR TS=("internists") OR TS=("nephrologist") OR TS=("nephrologists") OR TS=("neurologist") OR TS=("neurologists") OR TS=("neurosurgeon") OR TS=("neurosurgeons") OR TS=("oncologist") OR TS=("oncologists") OR TS=("ophthalmologist") OR TS=("ophthalmologists") OR TS=("otolaryngologist") OR TS=("otolaryngologists") OR TS=("physiatrist") OR TS=("physiatrists") OR TS=("pulmonologist") OR TS=("pulmonologists") OR TS=("radiologist") OR TS=("radiologists") OR TS=("radiotherapist") OR TS=("radiotherapists") OR TS=("rheumatologist") OR TS=("rheumatologists") OR TS=("surgeon") OR TS=("surgeons") OR TS=("traumatologist") OR TS=("traumatologists") OR TS=("urologist") OR TS=("urologists")

#2 TS=("absenteeism") OR TS=("disability management") OR TS=("employability") OR TS=("employable") OR TS=("employment") OR TS=("occupation") OR TS=("occupations") OR TS=("return to work") OR TS=("sick leave") OR TS=("sickness absence") OR TS=("unemployed") OR TS=("unemployment") OR TS=("vocational") OR TS=("work ability") OR TS=("work activity") OR TS=("work capacity") OR TS=("work disability") OR TS=("work participation") OR TS=("work rehabilitation") OR TS=("work retention") OR TS=("work status") OR TS=("workability") OR TS=("labo$r market entry")

#3 TS=("advice") OR TS=("advising") OR TS=("assisting") OR TS=("consultation") OR TS=("consultations") OR TS=("Counseling") OR TS=("Decision Making") OR TS=("discussing implications") OR TS=("discussing options") OR TS=("guidance") OR TS=("inform") OR TS=("information") OR TS=("Patient-Centered Care") OR TS=("recommendation") OR TS=("recommendations") OR TS=("refer") OR TS=("referral") OR TS=("support")

#1 AND #2 AND #3

limit to Dutch and Englisch and 2005-2021
