# Supplementary material for: Clinical Work-Integrating Care in Current Practice: A Scoping Review
Source: J Occup Rehabil. 2023 Nov 15;34(3):481–521. doi: 10.1007/s10926-023-10143-1 (PMC11364593; doi:10.1007/s10926-023-10143-1)
Supplement: Supplementary file 2 — Supplementary file2 (DOCX 13 KB) [file 10926_2023_10143_MOESM2_ESM.docx]

## Supplementary file 2 List of international gray literature sources

- Eurofound, <https://www.eurofound.europa.eu>, searched on 11-2-2022
- European Agency for Safety and Health at Work (EU-OSHA), <https://osha.europa.eu>, searched on 26-1-2022
- International Commission on Occupational Health (ICOH), <http://www.icohweb.org/site/homepage.asp>, searched on 11-2-2022
- International Labour Organization (ILO), <https://www.ilo.org/global/lang--en/index.htm>, searched on 14-2-2022
- Guidelines International Network (GIN), <https://g-i-n.net/international-guidelines-library/>, searched on 31-1-2022
- World Health Organization (WHO), <https://www.who.int>, searched on 21-1-2022
