# Supplementary material for: Clinical Work-Integrating Care in Current Practice: A Scoping Review
Source: J Occup Rehabil. 2023 Nov 15;34(3):481–521. doi: 10.1007/s10926-023-10143-1 (PMC11364593; doi:10.1007/s10926-023-10143-1)
Supplement: Supplementary file 3 — Supplementary file3 (DOCX 69 KB) [file 10926_2023_10143_MOESM3_ESM.docx]

|  | **First author and year** | **Country** | **Study type** | **Medical specialty** | **Study aim reported by author** | **Setting and recruitment** | **Study design** | **Study sample and size^a^** | **Response rate** | **Comment** |
| --- | --- | --- | --- | --- | --- | --- | --- | --- | --- | --- |
| 1 | Alexander 2012 | UK | Cross-sectional | All medical specialties, including general practice, not further specified | to (i) assess the amount and type of prior training received by hospital doctors, (ii) ascertain the confidence of individuals in writing the Fit note, Med 10 and the DS1500 and (iii) identify training needs | All trainee doctors form Plymouth Hospitals NHS Trust were invited | Two cycles of questionnaires to two different samples. Second cycle was sent after an e-learning module.  Study period: July 2011 and November 2011 | Trainee doctors including general practice vocational trainees (GPVTS), foundation years (FYs) one and two, core trainees (CTs) year one and two and all specialty registrar’s (SpRs) three to eight.  *n = 40, total sample of the first cycle*  n = 23, FYs,  n = 13, CTs  n = 2, GVTS,  n = 2, SpRs  n = 36, total sample of the second cycle  n = 14, FYs  n = 7, CTs  n = 1, GVTS  n = 14, SpRs | 13%, first cycle  12%, second cycle | Data extracted only from first cycle, data of interest not presented by the authors for the second cycle |
| 2 | Allen 2010 | UK | Cross-sectional | 23 medical specialties, not further specified | to investigate the attitudes and behaviors of hospital-based specialty training-grade registrars to the management of work and health issues and their sickness certification practices | All training-grade registrars from two UK deaneries (London and Wales) were invited | Questionnaire  Study period: 3-month period in 2009 | Secondary care training grade doctors on core training and specialist training  *n = 918, total sample* | 13-16% |  |
| 3 | Arrelöv 2007^b^ | Sweden | Cross-sectional | Orthopedic surgery, primary care | to explore perceived problems, and coping strategies related to sickness certification among General Practitioners and Orthopedic Surgeons | All physicians in two counties in Sweden (Stockholm and Östergötland) were invited | Questionnaire, subgroup analyses of orthopedic surgeons and general practitioners who handled sick-listing tasks at least a few times per year  Study period: October 2004 | Swedish physicians  n = 5455, total sample  *n = 149, orthopedic surgeons*  n = 673, general practitioners | 71.2%, of total sample |  |
| 4 | Bailey 2019 | USA | Qualitative | Hepatology | to investigate the difficulties inherent in affecting behavior change in patients  undergoing treatment using the Adaptive Leadership for Chronic Illness Framework | Patients and health care providers from a liver clinic of a university medical center serving North Carolina, USA, and a private, multi-specialty health care practice serving rural eastern North Carolina | In-depth interviews with patients. Semi-structured interviews with providers to elicit descriptions of a clinical encounter, added with recording and medical record from this encounter.  Study period: not reported | Chronic Hepatitis C patients and their health care providers *n = 11, patients* n = 10, health care providers  *n = 5, medical doctors*  n = 3, physician assistants  n = 2, nurse practitioners | N.A. |  |
| 5 | Bains 2012 | UK | Qualitative | Oncology | to explore the extent to which health professionals involved with colorectal cancer patients address work matters during active treatment | Health care professionals from UK National Health Service (NHS) trusts | Semi-structured interviews  Study period: January - June 2009 | Health care professionals treating employed colorectal cancer patient, either post-surgery or under active treatment  n = 18, total sample size  *n = 5, consultant surgeons*  *n = 3, oncologists*  n = 3, specialist nurses  n = 3, occupational health physicians  n = 3, general practitioners  n = 2, occupational health advisor / nurse | N.A. |  |
| 6 | Barber 2007 | UK | Cross-sectional | Pulmonology | to assess the approach to the diagnosis and management of occupational asthma amongst general (non-specialist) respiratory consultants in the UK | General respiratory physicians from NHS hospitals in UK were approached | Structured interview (face-to-face or telephone) or self-administered postal questionnaires using a case scenario of possible occupational asthma  Study period: April 2004 – January 2005 | General respiratory physicians without a specialist interest in occupational lung disease  *n = 45, total sample size*  n = 42, structured interviews  n = 10, questionnaires  n = 7, exclusions after data  collection | 42%, of total sample  21%, of sent questionnaires | Some key findings derived from discussion |
| 7 | Bardgett 2016 | UK | Qualitative | Orthopedic surgery | (i) to identify why certain factors may influence a total knee replacement patient’s return to work, and (ii) to identify potential deficiencies in the delivery of care directly pertinent to return to work | Patients selected form a cohort of 50 total knee replacement patients recruited into a population-based postal questionnaire study | Semi-structured one-on-one interviews  Study period: December 2013 - March 2014 | Total knee replacement patients who were preoperatively employed *n = 10, total sample size* | N.A. |  |
| 8 | Bayliss 2020 | UK | Cross-sectional | Cardiology, obstetrics and gynecology, oncology, orthopedic surgery | (i) to assess doctors’ attitudes to using patients’ occupation to guide clinical decisions and (ii) to evaluate training needs of doctors in asking about patients’ work | Hospital doctors working in a large teaching hospital in central London, UK | Questionnaire  Study period: December 2018 | Doctors working in cardiology, obstetrics and gynecology, oncology or orthopedics  *n = 42, total sample size*  n = 15, cardiology  n = 9, obstetrics and gynecology  n = 6, oncology  n = 12, orthopedics | 91%, of total sample |  |
| 9 | Bosma 2020 | the Netherlands | Qualitative | Not specified | to explore the lived experiences of Dutch workers with a chronic condition who are successful in staying at work, and identify facilitating factors, existing barriers and possible support needs for staying at work | Workers with a chronic condition purposeful selected after recruiting via newsletters of patient associations and social media | Four focus groups  Study period: August – December 2017 | Paid workers with chronic physical or mental conditions  *n = 30, total sample size* | N.A. |  |
| 10 | Bränström 2013^c^  Lindholm 2010^c^  Ljungquist 2013^c^ | Sweden | Cross-sectional | All medical specialties (including Primary health care; Internal medicine; Child and adolescent care; Surgery; Psychiatry; Gynecology; Orthopedics; Occupational health service; Ophthalmology; Ear, nose, and throat; Geriatrics; Infectious diseases; Oncology; Dermatology; Neurology; Rheumatology; Rehabilitation; Pain management; Other) | (i) to explore the frequency of sickness certifying for longer periods than actually would be necessary among physicians working in difference clinical settings; (ii) to examine main reasons for sickness certifying for longer periods than necessary; and (iii) to examine factors associated with unnecessary issued sickness certificates.  to gain detailed knowledge about physicians’ work with sickness-certification regarding frequency of different types of situations and severity of related problems, in general as well as in specific clinical settings  to explore to what extent physicians experienced sickness certification tasks as a psychosocial work environment problem and the possible associations of this with the type of clinic and some background and workplace factors | All physicians living and working in Sweden were invited | Questionnaire  Study period: October 2008 | Physicians working and living in Sweden  *n = 22 349, total sample size*  n = 14 210, physicians reporting handling sickness certification consultations at least a few times a year  n = 12 348, physicians reporting handling sickness certification consultations at least weekly  n = 4394, Primary health care  *n = 2121, Internal medicine*  n = 1665, Child and adolescent care  *n = 1562, Surgery*  n = 1284, Psychiatry  *n = 1070, Gynecology*  *n = 939, Orthopedics*  n = 500, Occupational health service  *n = 489, Ophthalmology*  *n = 486, Ear, nose, and throat*  n = 458, Geriatrics  *n = 342, Infectious diseases*  *n = 342, Oncology*  *n = 265, Dermatology*  *n = 259, Neurology*  *n = 193, Rheumatology*  *n = 190, Rehabilitation*  *n = 112, Pain management*  n = 3184, Other | 60.6%, of total sample |  |
| 11 | Bränström 2014^c^ | Sweden | Cross-sectional | Oncology | (i) to describe experiences concerning the sickness certification process, regarding problems and need for further competence concerning sickness certification; and (ii) to examine the association between experiences, perceived problems, need for education and issuing sickness certificates for longer periods than necessary. | All physicians living and working in Sweden were invited | Questionnaire, subgroup analyses of physicians working at oncology clinics  Study period: fall of 2008 | All physicians living and working in Sweden  n = 22 349, total sample size  *n = 348, physicians working mainly at oncology clinics* | 60.6%, of total sample |  |
| 12 | Braybrooke 2015 | UK, France, Germany | Cross-sectional | Oncology | to explore breast cancer patients’ personal experiences 1–5 years after AC treatment completion with changes in family life and relationship status, changes in working life and employment status and information about returning to work provided by healthcare professionals | Non-clinical setting through recruitment via mailing list form breast cancer advocacy groups and social media in UK, France and Germany. | Questionnaire  Study period: February - July 2012 | Breast cancer patients between 18 and 65 years *n = 198, total sample of which 31.8% UK, 38.9% French, 29.3% from German* | N.A. |  |
| 13 | Clayton 2007 | UK | Cross-sectional | Gynecology, Orthopedic surgery, Primary care, Occupational health care | to explore what advice is currently being given to patients by health care practitioners in respect of two common surgical procedures | (1) Occupational health physicians and general practitioners via two national conferences  (2) Consultant obstetricians and gynecologists within Sandwell and West Birmingham NHS Trust  (3) Consultant orthopedic surgeons at the Birmingham Orthopedic Hospital | Questionnaire and online available ‘patient information sheets’  Study period: March and April 2005 | Occupational Health physicians, General Practitioners, consultant gynecologists, consultant orthopedic surgeons.  n = 100, total sample size  n = 87, relating to BAH procedures  n = 38, OHPs  n = 38, GPs  *n = 11, consultant gynecologists*  n = 89, relating to BHR  n = 38, OHPs  n = 38, GPs  *n = 13, consultant orthopedic* surgeons  *n = 10, leaflets relating to BAH*  *n = 11, leaflets relating to BHR* | 6 %, of total sample  38 %, of OHPs  41 %, of GPs  100 %, of specialist consultants |  |
| 14 | Clinch 2009 | USA | Prospective cohort | Obstetrics | to provide an initial description of patient-provider communication surrounding women’s postpartum RTW | Postpartum women from a single mother-infant hospital unit in Forsyth County, NC | Interviewer-administered survey questionnaires. Measures were obtained at baseline (baby 4-months old) and at follow-up (baby 8-months old).  Study period: not reported | Postpartum women (age 18 and older) who had already returned or planned to return to full-time work (>30 hours per week) outside the home by the time their children were 4 months old, and had infants younger than 4 months. *n = 217, at baseline* | N.A. |  |
| 15 | Coole 2019 | UK | Qualitative | Orthopedic surgery, primary care | to explore the views and experiences of clinicians in treating working patients undergoing total hip or knee replacement. | Clinician working in primary and secondary National Health Service care in three areas of England (East Midlands, North East and Eastern regions) | Semi-structured interviews | Clinical stakeholder groups involved in treating employed patients undergoing Total Hip Replacement/Total Knee Replacement  *n = 40, total sample size*  n = 16, general practitioners  *n = 12, orthopedic surgeons*  n = 5, occupational therapists  n = 4, physical therapists  n = 3, nurses | N.A. |  |
| 16 | de Croon 2005 | the Netherlands | Cross-sectional | Rheumatology | (i) to examine associations between fatigue, psychosocial work characteristics, and physical work requirements on the one hand and self-reported work ability on the other, and (ii) to describe advice that health care professionals give to employees with RA on how to maintain their work ability | Patients part of cohort (1995-2000) from one single outpatient clinic for rheumatology and rehabilitation | Telephone interviews and self-reported questionnaires  Study period: 2001 | Newly referred patient with rheumatoid arthritis and being in paid employment at the start of the study *n = 78, total sample* | 98.7%, of total sample |  |
| 17 | Decuman 2015 | Belgium | Qualitative | Rheumatology | to describe, from the patient’s point of view, the factors influencing the occupational trajectory of patients with systemic sclerosis | Patients with systemic sclerosis in Belgium | Semi-structured interviews  Study period: not reported | Patients with systemic sclerosis *n = 14, total sample size* | N.A. |  |
| 18 | Dugan 2021 | USA | Qualitative | Oncology | (i) to identify the types of work-related support that are most valued and desired by breast cancer survivors as well as the specific types of support that were or were not received by survivors  (ii) to identify which healthcare providers were sources of support | Patients with breast cancer via flyers distributed at two cancer centers, breast cancer support groups, cancer events, cancer-related community-based organizations and a public university | Analysis of open-ended questions from a questionnaire  Study period: May 2017 - October 2018 | Patients who completed active primary treatment for breast cancer within the past 36 months and were employed at time of diagnosis  n = 143, total sample size  *n = 76, participants responded to open-ended questions* | 93.4% of eligible participants  53.1% of total sample responded to open-ended questions |  |
| 19 | Fowler 2019 | Australia and New Zealand | Cross-sectional | Colorectal surgery | to assess the consensus of current colorectal specialist practice in Australia and New Zealand with recent clinical practice guidelines for the management of hemorrhoids | All members of the Colorectal Surgical Society of Australia and New Zealand | Questionnaire using clinical based scenarios  Study period: not reported | Surgeons who are members of the Colorectal Surgical Society of Australia and New Zealand  *n = 82, total sample size* | 40% |  |
| 20 | Frank 2018 | Sweden | Qualitative | Cardiology | to investigate how healthcare professionals perceive collaboration and their work with patients of working age with heart failure | Health care professionals working in three cardiac clinics at urban located hospitals | Two focus groups and one individual interview  Study period: 2012 | Health care professionals working in health failure teams  *n = 8, total sample size*  n = 6, registered nurses  n = 1, physiotherapist  n = 1, cardiologist | N.A. |  |
| 21 | Gaudet 2019^e^  Rowe 2018^e^ | Canada | Prospective cohort | Emergency medicine | to document the occurrence of work-related concussion presenting to an emergency department for treatment  to determine whether structured discharge instructions and educational advice about the symptoms, sequelae, and normal progression of concussion provided by ED staff to adult patients seen in a Canadian ED for concussion decreases symptom persistence and time to return to usual activities | Patients presenting at emergency department of one academic hospital and two community hospitals | Semi-structured interview at presentation on emergency department, further data collection form the patient chart, electronic information system data, a questionnaire by the treating physician. Follow up at 30 and 90 days by standardized telephone interview.  Study period: April 2013 - April 2015 | Patients with Mild Traumatic Brain Injury, Glasgow Coma Scale 13-15, ≥17 years old  *n = 250, at baseline*  *n = 197, employed patients at baseline*  *n = 222 at 30 days follow up*  *n = 36 at 90 days follow up*  *n = 172, at least one follow up of employed patients* | N.A. |  |
| 22 | Grevnerts 2018 | Sweden | Cross-sectional | Orthopedic surgery, physical therapy | (i) to study and compare the factors Swedish orthopedic surgeons and physical therapists consider important for recommending anterior cruciate ligament reconstruction (ACL-R), and (ii) to assess how important orthopedic surgeons and physical therapists consider their own and each others’, as well as patients’, roles are in the treatment decision. | (1) Orthopedic surgeons registered in the Swedish national knee ligament register.  (2) Physical therapist from the Swedish Society for Physical Activity and Sports Medicine, Swedish Football Physiotherapists Association, orthopedic clinics over Sweden and through snowball recruitment | Questionnaire  Study period: April 2014 | Orthopedic surgeons and Physical therapist active in treating patients with ACL injury.  *n = 98, orthopedic surgeons*  n = 391, physical therapists | 46.7%, of orthopedic surgeons  50.0%, of physical therapist |  |
| 23 | Grewal 2014 | UK | Cross-sectional | General surgery | to assess what advice is currently being given to patients by surgeons after inguinal hernia repair in England. | Acute trusts in England were contacted | Survey and information extraction form leaflets  Study period: May 2012 | Acute trusts providing inguinal hernia repair *n = 128, total sample of acute trusts n = 93, leaflets returned* | 89.5%, of total sample  65% of acute trusts returned a leaflet |  |
| 24 | Gustavsson 2013^b,c^  Gustavsson 2016^c,d^ | Sweden | Cross-sectional | Gynecology / Obstetrics | (i) to explore frequencies of sickness certification consultations among gynecologists and obstetricians (O/Gs) and (ii) their experiences regarding severity of problems in these consultations, (iii) their organizational support and (iv) perceived need to acquire more competence regarding sickness certification issues | (1) All physicians in two counties in Sweden (Stockholm and Östergötland) were invited  (2) All physicians living and working in Sweden were invited  (3) All physicians in clinical settings where sickness certification occurred in Sweden were invited | Questionnaire send in 2004 (1), 2008 (2) and 2012 (3). Included in this article was a subgroup analysis of physicians mainly working in obstetrics, gynecology, or maternal health care.  Study period: (1) October 2004,  (2) October 2008, (3) October 2012 | Physicians working in gynecological, obstetrics or maternal health care.  *n = 315, physicians in O/Gs in 2004 questionnaire*  *n = 1037, physicians in O/Gs in 2008 questionnaire*  *n = 992, physicians in O/Gs in 2012 questionnaire* | 71%, of total study sample in 2004  66.9%, of physicians in O/Gs in 2008  63.6%, of physicians in O/Gs in 2012 |  |
| 25 | Hayman 2021 | Canada | Cross-sectional | Emergency medicine | to determine (i) what impacts sick notes have on patients and the system, (ii) the duration of time off work that physicians recommend, and (iii) what training and policies are in place to help providers | All physician members of the Canadian Association of Emergency Physicians | Questionnaire  Study period: December 2019 - January 2020 | Physicians working in emergency medicine  *n = 182, total sample size* | 11.9% |  |
| 26 | Hollick 2020 | UK | Mixed-methods | Rheumatology | to examine differences in clinical and patient-reported outcomes, including work outcomes, in individuals with axial spondyloarthritis living in rural and urban settings, using a sequential, explanatory mixed- method design | Patients selected from a cohort study from 83 centers across Great Britain | Mixed-methods using patient-reported outcomes with a questionnaire and semi-structured telephone interviews  Study period: | Patients with axial spondyloarthritis commencing biologic therapy at baseline  n = 2390, total sample size  n = 30, participants in interviews | N.A. |  |
| 27 | Holmlund 2021 | Sweden | Qualitative | Rehabilitation | to generate knowledge about how professional stakeholders organize and experience the support of the RTW process for persons with spinal cord injury (SCI) | Stakeholders who assisted persons with SCI in RTW purposeful sampled in three Swedish regions | Seven focus group conducted in five interview phases.  Study period: not reported | Stakeholders who have assisted the persons with SCI in the RTW process  n = 34, total sample size  n = 14, SCI rehabilitation team  *n = 4, physicians*  n = 3, occupational therapists  n = 2, physiotherapists  n = 4, social workers  n = 1, nurse  n = 5, Swedish Social Insurance Agency  n = 10, Swedish Public Employment Office  n = 6, caseworkers  n = 4, special consultants  n = 5, employers  n = 3, private sector  n = 1, municipality  n = 1, county council | N.A. |  |
| 28 | Holness 2007 | Canada | Cross-sectional | Pulmonology, primary care | to document and compare the knowledge and practice patterns related to occupational asthma, the barriers to early recognition, and educational needs for family physicians and pulmonologists | (1) All pulmonologist in Ontario (2) A random sample of 600 family physicians in Ontario | Questionnaire  Study period: not reported | Pulmonologist or family physicians. *n = 65, pulmonologists* n = 107, family physicians | 49%, of pulmonologists  26%, of family physicians |  |
| 29 | Jauregui 2020 | USA | Retrospective cohort | Emergency medicine | (i) to determine whether emergency department providers are following evidence-based low back pain management guidelines through the measurement of opioid versus NSAID prescribing for acute low back pain in the emergency setting and (ii) to identify whether any differences exist between demographic factors and prescribing habits of providers | Medical patients charts of a rural community hospital in the mid-Atlantic region of the USA | Retrospective chart review  Study period: January 2017 - June 2017 | Patients with new onset low back pain that started within the previous 4 weeks  n = 162, total sample size | N.A. |  |
| 30 | Jenny 2016 | France | Retrospective cohort | Orthopedic surgery | to report the functional outcome following anterior cruciate ligament (ACL) reconstruction in patients who decide when to resume work and normal sporting activity post-operatively. | Patients from a single orthopedic center | Retrospective analysis of prospectively collected data. Data collection was pre-operative, and at 6 weeks, 3 months, 6 months and 1 year follow up.  Study period: 2007 - 2012 | Patients undergoing primary ACL reconstruction *n = 72, at baseline n = 72, at 1 year follow up* | N.A. | Key findings derived from methods |
| 31 | Keegel 2007 | Australia | Cross-sectional | Dermatology | (i) to compare treatment and referral practices between general practitioners (GPs) and dermatologists (ii) to evaluate predictors for occupational contact dermatitis (OCD) disease severity measured in terms of worker impairment | (1) Clinicians from general practices, private dermatology practices and dermatology outpatient clinic attached to a public hospital in Dandenong, Australia were invited to report each worker with suspected OCD (2) Workers who attended a tertiary referral occupational dermatology clinic in Melbourne, Australia | Information was collected from case report forms, these patients were invited for assessment by a specialized occupational dermatologist to confirm OCD. Data was added via recruitment method (2) for the second study aim.  Study period: September 2002 - September 2003 | Workers suspected with occupational contact dermatitis n = 181, total sample size  n = 123, from recruitment method 1  n = 79, case reports forms from GPs  *n = 44, case reports forms from dermatologists*  n = 75, workers attended assessment from recruitment method 1 n = 58, from recruitment method 2 | 9%, of sample via recruitment method 1 |  |
| 32 | Kirchhoff 2017 | USA | Cross-sectional | Oncology | to survey providers regarding (i) their awareness of their adolescents and young adults (AYA) cancer patients’ unmet needs for financial support (e.g., insurance), treatment and clinical support (e.g., fertility care), and social and family support (e.g., mental health needs), as well as (ii) their knowledge of and referral practices to supportive care resources for their AYA cancer patients | Oncologists working in Utah identified from the two largest oncology providers and supplemented the Utah Department of Professional Licensing lists, the Society of Utah Medical Oncologists membership lists, and information from clinic websites. | Questionnaire  Study period: not reported | Oncologists (pediatric, medical, surgical, and radiation) providing medical services for cancer patients within the past year in Utah. *n = 91, total sample* | 57.6%, of total sample |  |
| 33 | Kosny 2018^e^  Russel 2019^e^ | Canada | Qualitative | Primary care, surgery, psychiatry, anesthesiology, oncology, rehabilitation, occupational health, and industrial, sports medicine | to understand Health care professionals’ experience with the WCB in their provinces and their role in the RTW process  to provide insight into strengthening stakeholder communication and collaboration, and facilitating sustainable rehabilitation and return-to-work for patients with work-related injuries | Health care providers working in primary and secondary care in four provinces in Canada (Ontario, British Columbia, Manitoba, and Newfoundland and Labrador) in large city centers and medium-sized centers and small cities and towns | Semi-structured one-on-one interviews  Study period: May - November 2015 | Health care professionals who had seen at least one patient with a work injury in the previous year  n = 131, total sample size  n = 59, general practitioners  *n = 19, medical specialists*  n = 19, allied healthcare providers  n = 34, case managers | N.A. |  |
| 34 | Ladak 2021 | Canada | Cross-sectional | Rheumatology, dermatology, gastroenterology | (i) to assess whether Canadian rheumatologists, dermatologists and gastroenterologists are being asked to provide advice on returning to work amid the COVID-19 pandemic and how they approach these clinical encounters  (ii) to assess whether these physicians would provide a medical note for delayed return-to-work or modified duties | Physicians registered with the Canadian  Rheumatology Association (CRA), Canadian Dermatology  Association (CDA) and Canadian Association of Gastroenterologists (CAG) via e-mail invitation, electronic newsletter and social media | Questionnaire, including seven clinical scenarios  Study period:  September 2020 | Canadian rheumatologists, dermatologists or gastroenterologists  n = 151, total sample size  n = 125, completed questionnaires  n = 26, partially completed questionnaires  n = 92, rheumatologists  n = 45, gastroenterologists  n = 14, dermatologists | not reported |  |
| 35 | Lamort-Bouché 2021 | France | Qualitative | Oncology | (i) to explore the perspective of breast cancer specialists regarding their perceived professional role toward RTW needs of their breast cancer patients  (ii) to identify barriers and facilitators for teamwork between breast cancer specialists and general practitioners and occupational physicians in the RTW process | Breast cancers specialists in the urban area of Lyon purposeful recruited through snowball sampling | Semi-structured one-on-one interviews  Study period: not reported | Breast cancers specialists  *n = 20, total sample size*  n = 8, medical oncology  n = 6, radiation oncology  n = 6, gynecological surgery | N.A. |  |
| 36 | Ljungquist 2015^d^ | Sweden | Cross-sectional | All medical specialties (including Primary health care; Internal medicine; Surgery; Psychiatry; Gynecology/Obstetrics; Orthopedics; Oncology; Occupational health service; Infectious diseases; Neurology; Dermatology; Rheumatology; Rehabilitation; Pain management) | to explore how problematic physicians in different clinical settings experience sickness certification tasks in general as well as regarding specific issues related to assessment of function, work capacity, and need for sick leave | All physicians working and living in Sweden. Participants were identified by a company that manages registries of health care staff | Questionnaire, subgroup of physicians who had consultations concerning sickness certification at least a few times a year  Study period: October 2012 | Physicians working and living in Sweden who had consultations concerning sickness-certification at least a few times a year.  n = 12933, total sample size  n = 4088, Primary health care  *n = 1756, Internal medicine*  *n = 1333, Surgery*  n = 993, Psychiatry  *n = 877, Gynecology/Obstetrics*  *n = 864, Orthopedics*  *n = 346, Oncology*  n = 336, Occupational health service  *n = 322, Infectious diseases*  *n = 252, Neurology*  *n = 176, Dermatology*  *n = 182, Rheumatology*  *n = 130, Rehabilitation*  *n = 65, Pain management*  n = 1147, Other | 57.6%, of total sample |  |
| 37 | Löfgren 2007^b^ | Sweden | Cross-sectional | All medical specialties (including Primary health care; Internal medicine; Psychiatry; Surgery; Gynecology / Obstetrics; Orthopedics; Occupational health; Oncology; Rehabilitation care; Addiction medicine) | to study physicians’ sickness certification practices with regard to the frequency of consultations involving consideration of sickness certification and frequency and nature of problems experienced related to this task, in general and in different clinics/practices | All physicians in two counties in Sweden (Stockholm and Östergötland) were invited | Questionnaire, subgroup analyses of physicians who had consultations concerning sickness certification at least a few times a year  Study period: October 2004 | Physicians  n = 5455, total sample size  n = 4019, handling sick-listing at least a few times a year  n = 978, Primary health care  *n = 396, Internal medicine*  n = 394, Psychiatry  *n = 218, Surgery*  *n = 215, Gynecology / Obstetrics*  *n = 200, Orthopedics*  n = 124, Occupational health  *n = 108, Oncology*  *n = 75, Rehabilitation care*  *n = 47, Addiction medicine*  n = 2368, Other | 71%, of total sample |  |
| 38 | MacLennan 2017 | UK | Qualitative | Urology / Oncology | to develop a better understanding of the context within which those diagnosed with urological cancer experienced return to work or continuation of work, adapted to work and were informed and advised on working | Members of the three stakeholder groups across North East Scotland | Semi-structured one-on-one interviews in person or by telephone  Study period: not reported | (1) working men and women diagnosed with bladder, kidney or prostate cancer within the last 5 years; (2) health and care providers in secondary care specializing in cancer, and (3) professional staff and line managers from large employing organizations n = 32, total sample size  *n = 12, individuals with urological cancer*  n = 10, specialist health and care providers  n = 5, urological cancer nurse specialists  *n = 3, consultant urologists  n = 2, consultant clinical oncologists*  n = 10, staff members of large employing organizations | N.A. |  |
| 39 | Main 2005 | USA | Qualitative | Oncology | to describe work issues and work return among a diverse group of cancer survivors who were working at the time of diagnosis | Cancer patients registered in the Colorado Central Cancer Registry using stratified sampling | Semi-structured face-to-face interviews  Study period: not reported | People between the ages of 21 and 66 years, who were working in a paid job prior to the cancer diagnosis *n = 28, total sample size* | N.A. |  |
| 40 | Meunier 2016 | France | Cross-sectional | Rheumatology | to ascertain how often French rheumatologists and RA patients discuss work during consultations | All French rheumatologist from the French National Medical Association. Patients independently recruited via pharmacies | Questionnaire administered by telephone to rheumatologists and self-administered by patients. Rheumatologist were asked to describe their most recent consultation with an RA patient and vice versa.  Study period: 2011 | Rheumatologists who had consultations with a patients with rheumatoid arthritis (RA) <1 month ago, age between 20 and 59, with a job or unemployed but looking for a job, and RA patients with the above criteria *n = 153, rheumatologists n = 81, RA patients* | 9%, of rheumatologist  N.A. for patients |  |
| 41 | Michel 2018 | France | Cross-sectional | Rehabilitation | (i) to assess the tools and methods used for the collection of workplace information by rehabilitation centers and (ii) to explore the information sharing between centers’ practitioners and occupational health professionals for the management of chronic low back pain patients | Rehabilitation centers in France were contacted | Questionnaire sent to managers of functional restoration program  Study period: April 2014 | French rehabilitation centers offering a functional restoration program to chronic low back pain patients *n = 56, total sample size* | 95.7%, of total sample |  |
| 42 | Mirmohammadi 2013 | Iran | Prospective cohort | Cardiology | to assess the frequency of return to work after the first myocardial infarct, and its influencing factors | Single cardiac department | Questionnaire at 6-months after first myocardial infarct and one year follow-up  Study period: September 2007 - September 2010 | Patients with first myocardial infarct who were employed before MI. *n = 200, at baseline n = 174, at 1 year follow up* | N.A. |  |
| 43 | Morrison 2015 | Canada | Qualitative | Oncology, primary care | to explore physicians’ perspectives on supporting cancer survivors’ work integration issues | Oncology team at one large oncology center and family physicians from a wider network | Semi-structured one-on-one interviews  Study period: not reported | Physicians with an active clinical role with cancer survivors *n = 10, total sample size  n = 5, oncologists*  n = 5, family physicians | N.A. |  |
| 44 | Moscato 2014 | Italy | Cross-sectional | Allergology | to evaluate the awareness of occupational asthma among Italian allergists | Members of the Association of Italian Allergists via mail invitation | Web-questionnaire  Study period: 11 April - 11 July 2014 | Members of the Italian Association of Allergists *n = 14, total sample size n = 80, number of cases reported by allergists* | 2.4%, of total sample |  |
| 45 | Naidu 2012 | UK | Cross-sectional | Gynecology and obstetrics | to establish the current practice in terms of advice given for activities during convalescence after commonly performed benign gynecological surgery by obstetricians and gynecologists in the UK | All obstetrician and gynecologists in the UK identified from the National Health Service (NHS) website | Questionnaire  Study period: not reported | Obstetricians and gynecologists *n = 472, total sample size* | 30.5%, of total sample |  |
| 46 | Newington 2018 | UK | Cross-sectional | Hand surgery, occupational and physical therapy | to investigate the RTW recommendations of practicing clinicians for patients undergoing carpal tunnel release (CTR) | Members of the British Society for Surgery of the Hand, the Association of Surgeons in Primary Care, the Reconstructive Surgical Trials Network and the British Association of Hand Therapists | Questionnaire  Study period: November - December 2016 | Surgeons and therapists treating CTR patients who were workers in the past 12 months n = 310, total sample size  *n = 173, surgeons*  n = 137, therapists | 13.5%, of surgeons 20.1%, of therapists |  |
| 47 | Newington 2019 | UK | Qualitative | Hand surgery, rheumatology | (i) to explore patients’ perspectives of returning to their work after carpal tunnel release (CTR) and (ii) to identify the factors that influenced this return to work experience | Patients selected from a cohort study from 16 sites across England and Wales | Semi-structured one-on-one interviews  Study period: August 2017 - June 2018 | Patients who have had CTR and who had paid employment with the intention to RTW prior to the operation  *n = 14, total sample size* | N.A. |  |
| 48 | Nilsing 2014 | Sweden | Cross-sectional | Primary health care, Occupational health services, Private clinics, Hospital care | (i) to investigate whether patients are prescribed rehabilitation early in a new sick leave period, and (ii) whether the prescription is associated with patient’s sex, age, diagnosis, description of functioning and affiliation of the certifying physician | Sickness certificates delivered to social insurance offices in Östergötland County, Sweden | Information collected from sickness certificates delivered to social insurance offices during two weeks and followed until the end of the current sick leave period  Study period: September 2007 | All new sickness certificate during a two week period delivered to the social insurance offices n = 497, total sample size n = 470, sample size with known physician affiliation  n = 201, primary health care  n = 24, occupational health services  *n = 39, private clinics  n = 206, hospital* | N.A. |  |
| 49 | O'Hagan 2011 | Canada | Cross-sectional | Cardiac rehabilitation | (i) to assess the association between perceived support from family, physicians, and employers in relation to work status and self-reported work adjustment in CR patients initiating a CR program as well as those further along in rehabilitation (ii) to examine the concurrent associations of social support with job characteristics including job demand (physical and psychological) and job control | Patients from the Toronto Rehabilitation Institute cardiac rehabilitation program | Questionnaire  Study period: not reported | Patients from the Toronto Rehabilitation Institute CR program. Participants were working prior to their event or surgery and only those with the intention of returning to the workforce were included in the study; self-employed participants were excluded *n = 214, total sample size*  n = 126, at program entry  n = 88, at 6 to 12 months of participation | not reported |  |
| 50 | Paniccia 2019 | Canada | Qualitative | Rehabilitation | to provide insight on youth and young adults with acquired brain injury by examining young people’s experiences of, and transition towards work-related roles | Patients form a children’s rehabilitation hospital, a university campus (e.g., hospital and university research websites, student roster lists), and acquired brain-injury community support groups via posted recruitment flyers | Semi-structured interviews  Study period: September 2015 - May 2016 | Individuals aged 15-25 years with a self-reported, ABI diagnosis; enrolled or recently completed high school or college/university ( 6 months); and experiencing ongoing ABI-related symptoms. *n = 14, total sample size* | N.A. |  |
| 51 | Ratzon 2006 | Israel | Prospective cohort | Surgery | to determine factors predicting a delayed return to work | Patients from a single surgical department | Surgeon's recommendations were collected at baseline, functional status was determined pre-operatively and at 1 month post-operative, symptom severity and RTW status every two weeks by telephone up till 90 days.  Study period: not reported | Employed patients consecutively referred for carpal tunnel surgery, hospitalized and operated *n = 50, total sample size* | N.A. | Some key findings derived from methods |
| 52 | Salit 2020 | USA | Cross-sectional | Oncology | to determine (1) whether transplantation centers have guidelines for RTW post-Hematopoietic Cell Transplantation (HCT), and the consistency of these guidelines, and (2) whether transplantation centers have RTW programs for their patients, and the characteristics of these programs | National and international transplantation centers identified through the National Marrow Donor Program (NMDP)/Be The Match | Questionnaire  Study period: not reported | Transplantation centers performing >50 total (combined allogeneic and autologous) HCTs per year *n = 45, total sample size* | 30%, of total sample |  |
| 53 | Skudlik 2008 | Germany | Prospective cohort | Dermatology | to analyze the efficacy of the intensive interdisciplinary measures of tertiary individual prevention (TIP) | Patients who took part in TIP in a single academic hospital | Follow-up with a standardized questionnaire 1 year after discharge from the in-patient phase of the TIP program.  Study period: 1994 – 2003 | Patients with severe occupational skin diseases who were eligible for TIP (no successful out-patient treatment and threatened with job-loss) n = 1486, patients in TIP n = 1178, patient returning questionnaire | N.A. | Key findings derived from introduction and methods |
| 54 | Snöljung 2017^d^ | Sweden | Cross-sectional | Neurology | to describe experiences among specialists and non-specialists working in neurology clinics concerning their work with sickness certification of patients, regarding frequency of specific situations, perceived problems, need for competence, among all and among | Physicians working and living in Sweden, participants were identified by a company that manages registries of health care staff | Questionnaire, subgroup analysis of physicians mainly working in neurology clinics  Study period: October 2012 | Physicians living and working in Sweden  n = 19 223, total sample size  *n = 265, mainly working in neurology clinics (neurologists)* | 58%, of total sample size |  |
| 55 | Söderman 2021 | Sweden | Cross-sectional | Oncology | (i) to investigate oncologists’ experiences of organizational prerequisites for sickness certification tasks, and (ii) if lack of resources was related to experiencing sickness certification as problematic | All physicians living and working in Sweden were invited, participants were identified by a company that manages registries of health care staff | Questionnaire, subgroup analysis of physicians working in oncology or hematological clinics  Study period: October 2017 | Physicians living and working in Sweden  n = 18 714, total sample size  n = 351, physicians working mainly at oncology clinics (oncologists)  *n = 342, oncologists who stated that they had sickness certification consultations at least a few times per year* | 54.1%, of total sample  52.4%, of oncologists |  |
| 56 | Steenbeek 2014 | the Netherlands | Prospective longitudinal | Not specified | to explore workers’ opinions on the effect of contact with health care providers on sickness absence duration | Workers from an existing internet panel of a large market research organization | Three-wave study with an additional fourth questionnaire. The first three wave covered a period of 1.5 years, the fourth question was sent 1.5 years later to the same workers as in the first wave.  Study period: 2005 - 2008 | Workers with a weekly employment contract of > 12 hours and with health complaints, chronic disease and/or a recent history of sickness absence *n = 3048, at first wave n = 1424, respondents to all 4 measurements* | N.A. |  |
| 57 | Swartling 2008 | Sweden | Qualitative | Orthopedic surgery | to identify and describe the views among Swedish orthopedic surgeons on sick-listing practice and on the sick-listing commission, and to discuss this in comparison with general practitioners views | Five orthopedic clinics in four Swedish counties. | Semi-structured one-on-one interviews. Interview guide based on previous study of general practitioners  Study period: June – August 2004 | Orthopedic surgeons *n = 17, total sample size* | N.A. |  |
| 58 | Szekeres 2018 | UK | Qualitative | Orthopedic surgery, Plastic surgery | (1) to describe and characterize the decision-making process of the Hand and Upper Limb Centre Workplace Safety and Insurance Board (WSIB) surgeons when managing injured workers.  (2) to identify the methods and tools used by surgeons to assess and manage injured workers | Surgeons working in WSIB Upper Extremity Specialty Clinic at the Hand and Upper Limb Centre at St. Joseph’s Health Care, London | Semi-structured one-on-one interviews, followed by an observation by the same interviewer in a clinic visit during a new patient assessment in which additional field notes were made  Study period: not reported | Orthopedic surgeons or plastic surgeons *n = 9, total sample size* | N.A. |  |
| 59 | Takahashi 2018 | Japan | Cross-sectional | Oncology | to (i) reveal the present situation of survivors’ job resignation, its timing and reasons; (ii) examine healthcare providers’ screening behaviors of survivors’ work-related difficulties and (iii) reveal changes to survivors’ information/support needs according to time since diagnosis | Patients re-visiting outpatients at three cancer centers in Japan | Questionnaire  Study period: October – December 2015 | Patients with cancer who had paid work at time of diagnosis *n = 950, total sample size* | 87.6%, of total sample |  |
| 60 | Thompson 2013 | Australia | Mixed-methods | Oncology | (1) to explore multidisciplinary oncology health care professionals’ understanding of the preferences of young people living with cancer, receiving treatment at Peter Mac. (2) to identify any challenges faced by professionals within current practice and how these differ across professional disciplines. | Health care professionals working at Peter Mac Victorian Adolescent & Young Adult Cancer Service. | Questionnaire with both quantitative and qualitative sections | Health care professionals working at Peter Mac and working with AYA patients over the last 12 months. n = 60, total sample size  n = 7, AYA cancer service  *n = 6, oncologists*  n = 16, nurse, ward based  n = 6, nurse, chemotherapy day care  n = 10, clinical nurse consultant  n = 9, social work  n = 3, occupational therapy  n = 2, physiotherapy  n = 1, clinical psychology | 61%, of total sample |  |
| 61 | Tiedtke 2012 | Belgium | Qualitative | Oncology | (i) to elucidate the Flemish RTW state of affairs experienced by the stakeholders involved, and (ii) to better understand the relationship between RTW policy and practice in the case of employees with breast cancer | Stakeholders who attended a local, multidisciplinary breast cancer conference in the Province of Limburg (Flanders) | Three focus groups, carried out simultaneous at a conference  Study period: October 2009 | Stakeholders involved in the RTW process of breast cancer patients n = 26, total sample size  *n = 4, treating physicians*  n = 6, employers  n = 3, social security physicians  n = 4, occupational physicians  n = 5, 'hands-on' experts i.e. breast cancer survivors  n = 4, representatives of patient associations | N.A. |  |
| 62 | Tsang 2020 | UK | Cross-sectional | Orthopedic surgery | to establish who is delivering occupational advice, when and where it is being delivered and what advice is being offered | Clinician registered with the National Joint Registry for England, Wales and Northern Ireland and members of Scottish Committee for Orthopedics and Trauma were invited; link to survey embed in an article of a previous study | Questionnaire  Study period: July – August 2017 | Clinicians working in surgical teams offering hip and knee replacements  n = 152, total sample size  *n = 78, orthopedic surgeons*  n = 20, physiotherapists  n = 25, occupational therapists  n = 25, nurse / specialist nurse / extended scope practitioners  n = 4, other | Not reported |  |
| 63 | van der Meer 2011 | the Netherlands | Qualitative | Rheumatology | to investigate the experiences and needs of employees with rheumatoid arthritis treated with anti-TNF therapy with respect to work participation | Patients form the Rheumatology Outpatient Clinic of an academic medical center in  Amsterdam, the Netherlands, who were part of an ongoing cohort study | Semi-structured interviews  Study period: December 2008 - January 2009 | Employed patients with established rheumatoid arthritis treated with anti-tumor necrosis factor therapy *n = 14, total sample size* | N.A. |  |
| 64 | Van Velzen 2020 | the Netherlands | Cross-sectional | Rehabilitation | to provide insight into the availability and processes of VR services provided by medically specialized rehabilitation institutions in the Netherlands | ^1^Data extracted form sample displayed in italics  ^2^Part of large Swedish cross-sectional study in 2004  ^3^Part of large Swedish cross-sectional study in 2008  ^4^Part of large Swedish cross-sectional study in 2012  ^5^Both studies reported on the same data  Abbreviates: RTW - Return to Work; UK - United Kingdom; USA - United States of America | Questionnaire  Study period: not reported | Dutch medically specialized rehabilitation institutions  *n = 55, total sample size*  n = 15, institution providing both in- and outpatient rehabilitation  n = 36, institutions providing outpatient rehabilitation only  n = 4, institutions providing only consultations | 46% | One key finding derived from introduction |
| 65 | Wada 2012 | Japan | Cross-sectional | Oncology | to elucidate the awareness and behavior of oncologists in supporting patient employment, the current status of support in medical institutions and the association of the two | (1) all diplomats and faculty of the subspecialty board of medical oncology in the Japanese Society of Medical Oncology  (2) all surgeons who were certified by the Japanese Board of Cancer Therapy and living in the Kanto area | Questionnaire  Study period: 2010 - 2011 | Doctors working in medical oncology. *n = 668, total sample size*  n = 195, internal medicine  n = 357, surgery  n = 24, brain surgery  n = 24, radiation  n = 67, other | 45.5%, of total sample |  |
| 66 | Walker 2007 | UK | Cross-sectional | Emergency medicine | to assess whether emergency departments and fracture clinics in the UK are issuing sick notes in line with the guidance 'Making a difference: reducing general practitioner paperwork' | Emergency departments and fracture clinics in Scotland and England | Short survey  Study period: not reported | Hospitals with both accident and emergency and fracture clinics *n = 50, total sample*  n = 25, Scottish emergency hospitals  n = 25, English emergency hospitals | 100%, of total sample |  |
| 67 | Walters 2010 | UK | Cross-sectional | General surgery, internal medicine, emergency medicine, general practice, orthopedics | (1) to see what sickness certification training had been received by hospital postgraduate trainees and establish how confident and knowledgeable they were (2) to evaluate the feasibility and face validity of an on-the-job paper-based educational module. | Doctors in training in West Midlands Deanery posts | Structured interview with closed and open questions, and a 10 minute demonstration of a training module followed with a brief evaluation | Registered doctors in training in a variety of specialties at all grades from Foundation Year 2 to specialist registrar and practicing sickness certification *n = 51, total sample size* | not reported |  |
| 68 | Watson 2009 | USA | Cross-sectional | Orthopedic surgery | to investigate factors other than fracture union that determine medical clearance for work duty | Members of the American Society for Surgery of the Hand (ASSH) | Questionnaire  Study period: not reported | Orthopedic surgeons who were member of the ASSH *n = 125, total sample size* | not reported |  |
| 69 | Zegers 2020 | the Netherlands | Cross-sectional | Oncology | to explore: (i) the extent to which hospital  healthcare professionals conduct conversations about work-related issues with cancer survivors, (ii) whether CSs experience these conversations as helpful, and (iii) the possible financial implications for cancer survivors of (not) discussing their work early on | A link to the survey was distributed via social media and e-mailed to members of various cancer patient organizations and Dutch Federation of Cancer Patient Organizations patient panel | Questionnaire  Study period: February - March 2019 | Cancer survivors who were employed at time of diagnosis  *n = 3500, total sample size* | not reported |  |
| 70 | Zirkzee 2008 | the Netherlands | Prospective cohort | Rheumatology | (i) to describe the occurrence of sick leave and permanent work disability and its predictors in the very early phases of the disease (ii) to describe the provision of adaptations to the workplace and professional guidance in patients with chronic arthritis | Patients from the early arthritis clinic cohort from one academic hospital | Assessment at study entry and after 12 months.  Study period: 2000 - 2003 | Patients with rheumatoid arthritis with active disease, disease duration <2 years and having a paid job <12 months before the first visit of the clinic *n = 69, total sample size* | N.A. |  |
| ^a^Data extracted from sample displayed in italics; ^b^Part of large Swedish cross-sectional study in 2004; ^c^Part of large Swedish cross-sectional study in 2008; ^d^Part of large Swedish cross-sectional study in 2012; ^e^Both studies reported on the same data  Abbreviations: RTW - Return to Work; UK - United Kingdom; USA - United States of America | | | | | | | | | | |
